# Bacterial chromosomal gene positioning is likely shaped by selection on both mean and growth-dependent expression

**DOI:** 10.1371/journal.pgen.1012316

**Published:** 2026-09-21

**Authors:** Ruiqi Yuan, Jianzhi Zhang

**Affiliations:** Department of Ecology and Evolutionary Biology, University of Michigan, Ann Arbor, Michigan, United States of America; North Carolina State University, UNITED STATES OF AMERICA

## Abstract

In bacteria with circular chromosomes, genes near the replication origin (*oriC*) are replicated earlier and consequently attain higher copy numbers than genes near the terminus (*ter*), particularly during rapid growth. Hence, mutations altering a gene’s chromosomal position can affect its expression level and be subject to selection. Two non–mutually exclusive hypotheses regarding the target of this selection have been proposed. The mean expression hypothesis (MEH) posits that the target is a gene’s average expression across environments, whereas the growth-dependent expression hypothesis (GEH) proposes that the target is the growth dependence of gene expression, quantified by the expression slope—the change in expression level per unit change in growth rate. To test these hypotheses and assess their relative support, we analyze eight multi-environment protein expression datasets from three bacterial species, as well as *Escherichia coli* promoter strengths measured in two environments. Consistent with both MEH and GEH, we observe a significant decrease in both mean expression and expression slope from *oriC* to *ter* in six and four of the eight datasets, respectively. In regression models predicting gene position, the relative contributions of the two hypotheses differ across species. However, even when combined, the two hypotheses explain only a small fraction of chromosomal gene positioning, in part because the replication-dose effect is incompletely offset by compensatory evolution of individual promoter strengths. The positional gradients in mean and growth-dependent expression are disproportionately contributed by genes involved in translation and transcription. We conclude that patterns of bacterial chromosomal gene positioning are consistent with moderate effects of selection on both mean and growth-dependent expression.

## Author summary

Bacterial gene positioning affects gene expression because genes near the origin of chromosomal replication are replicated earlier than those near the terminus and thus have higher gene copy numbers, especially during rapid population growth. Consequently, mutations altering the chromosomal position of a gene influence its expression so could be targeted by natural selection. Two hypotheses propose that the selection target is the mean expression level of a gene across environments and the growth dependence of expression, respectively. This study analyzed multi-environment protein expression datasets from three bacterial species to assess the two hypotheses. We found evidence for both hypotheses, but also discovered that, even when combined, the two hypotheses explain only a small fraction of chromosomal gene positioning, in part because the replication-dose effect is incompletely offset by compensatory evolution of individual promoter strengths. We conclude that patterns of bacterial chromosomal gene positioning are consistent with moderate effects of selection on both mean and growth-dependent expression, but more bacteria should be studied in the future to test the generality of our findings.

## Introduction

Most bacterial genomes consist of a single circular chromosome [1]. During replication of the circular genome, two replication forks form at the chromosome replication origin (*oriC*) and proceed towards the terminus (*ter*). The two replichores produced in this process are typically symmetric and of the same size [2–4]. There can be more than two replication forks proceeding simultaneously in a bacterial cell during fast growth because a new round of replication can initiate before the previous one completes. The number of simultaneous replication rounds (*R*) can be estimated by the ratio of the time for replication forks to travel from *oriC* to *ter* (*C*) to the time interval between two successive cell divisions (i.e., doubling time *G*), given that exactly one round of replication is needed per cell cycle [1]. Because growth rate *μ* equals ln2/*G*, *R* = *C*/*G* = *Cμ*/ln2 [5].

Genes near *oriC* attain higher copy numbers than genes near *ter* during replication because the former are always replicated earlier than the latter. This replication-associated gene dosage disparity increases with growth rate, because a gene near *oriC* has a copy number that is 2^*R*^ = *e*^*Cμ*^ times that of a gene near *ter* [4]. The relative dosage of gene *X*, *D*_*X*_, is defined as the ratio of the average copy number of *X* to the average copy number of a hypothetical gene at the midpoint between *oriC* and *ter*. Based on the Cooper-Helmstetter model of bacterial chromosome replication [6,7], it can be shown that


$$\begin{array}{c} \text{ln}\;D_{X}=(\text{0}.\text{5}-x)C\mu \text{.} \end{array}$$ (1)

Here, *x* is the gene position normalized to the range of [0, 1], which is the distance from the gene’s midpoint to the midpoint of *oriC* divided by one half of the chromosome length.

A mutation altering a gene’s chromosomal position influences its dosage, which in turn influences its mRNA or protein expression level. Indeed, experimentally inserting a transgene near *oriC* resulted in a higher transgene expression than inserting it near *ter* in both *Salmonella typhimurium* and *Escherichia coli* [8–10]. Furthermore, empirical data have shown that expression levels of artificially translocated genes are approximately proportional to the gene dose predicted by the Cooper-Helmstetter model [8,9,11]. Hence,


$$\begin{array}{c} \text{ln}\;E_{X}=(\text{0}.\text{5}-x)C\mu \text{.} \end{array}$$ (2)

Here, $E_{X}$ is the relative expression level of gene *X* (i.e., expression level of *X* divided by that when it is located at the midpoint between *oriC* and *ter*). Although *C* is often assumed to be a constant at rapid growth [5,7], studies have reported that *C* increases as *μ* decreases during slow growth [12–14]. An empirical formula relating *C* with *μ* was obtained for *E. coli* by fitting experimental data [14]:


$$\begin{array}{c} C=\text{0}.\text{6}\text{1}\text{9}+\frac{\text{0}.\text{1}\text{7}\text{5}}{\mu }\text{.} \end{array}$$ (3)

Plugging Eq 3 into Eq 2 yields:


$$\begin{array}{c} \text{ln}\;E_{X}=(\text{0}.\text{6}\text{1}\text{9}\mu +\text{0}.\text{1}\text{7}\text{5})(\text{0}.\text{5}-x)\text{.} \end{array}$$ (4)

Eq 4 shows that, given μ, $\text{ln}\;E_{X}$ declines with *x*. Furthermore, as μ rises, $\text{ln}\;E_{X}$ increases more when *x* is close to 0 than when it is close to 0.5 and $\text{ln}\;E_{X}$ decreases more when *x* is close to 1 than when it is close to 0.5. That is, gene position is expected to influence both the level of gene expression and its growth dependence. Hereinafter, we define the replication-dose effect as the effect of chromosomal replication-associated gene dose change on gene expression. Note that the replication-dose effect increases with the growth rate. We refer to this growth-dependent property of the replication-dose effect as the growth-dependent replication-dose effect.

That altering the chromosomal position of a gene influences its expression prompted the hypothesis that chromosomal gene positioning is subject to expression-related selection [5,15–17]. In theory, gene positioning can be subject to purifying and positive selection. First, upon the optimization of gene expression, gene position should generally be subject to purifying selection and conserved. The observation that gene position is conserved across bacterial species [16] despite high rates of genome rearrangement [4,18] provides empirical evidence for purifying selection on gene position. Second, if gene expression is suboptimal, mutations relocating the gene could be advantageous. Empirical evidence for such adaptive gene relocation would support positive selection acting on gene positioning. However, such evidence is scarce. In Lenski’s Long-Term Evolution Experiment of 12 replicate *E. coli* populations, a large inversion in the same genomic region was found in three populations [19]. Although the repeated acceptance of this inversion could have been driven by positive selection on gene positioning, it could also be explained by positive selection for a switch of genes from the lagging to the leading strand that could prevent head-on collisions between DNA and RNA polymerases that are particularly deleterious [20].

To explore potential positive selection on gene positioning at the chromosomal scale, let us consider the following scenario. Genes are initially randomly thrown to a chromosome. So, their expression levels, which are jointly determined by their chromosomal positions and transcriptional regulations, are suboptimal. Optimal gene expression levels may be achieved by (i) beneficial mutations changing gene positions and/or (ii) beneficial mutations changing transcriptional regulations, especially promoter strengths. If only (i) occurs, the outcome would be a trend for the optimal gene expression level to decrease from genes near *oriC* to genes near *ter* to take advantage of the replication-dose effect. If only (ii) occurs, the outcome would be a trend for the promoter strength to increase from genes near *oriC* to genes near *ter*, to offset the replication-dose effect. If both (i) and (ii) occur, both outcomes may be observed but with weakened trends. A test of these predictions can help infer adaptive gene positioning.

**Fig 1:**
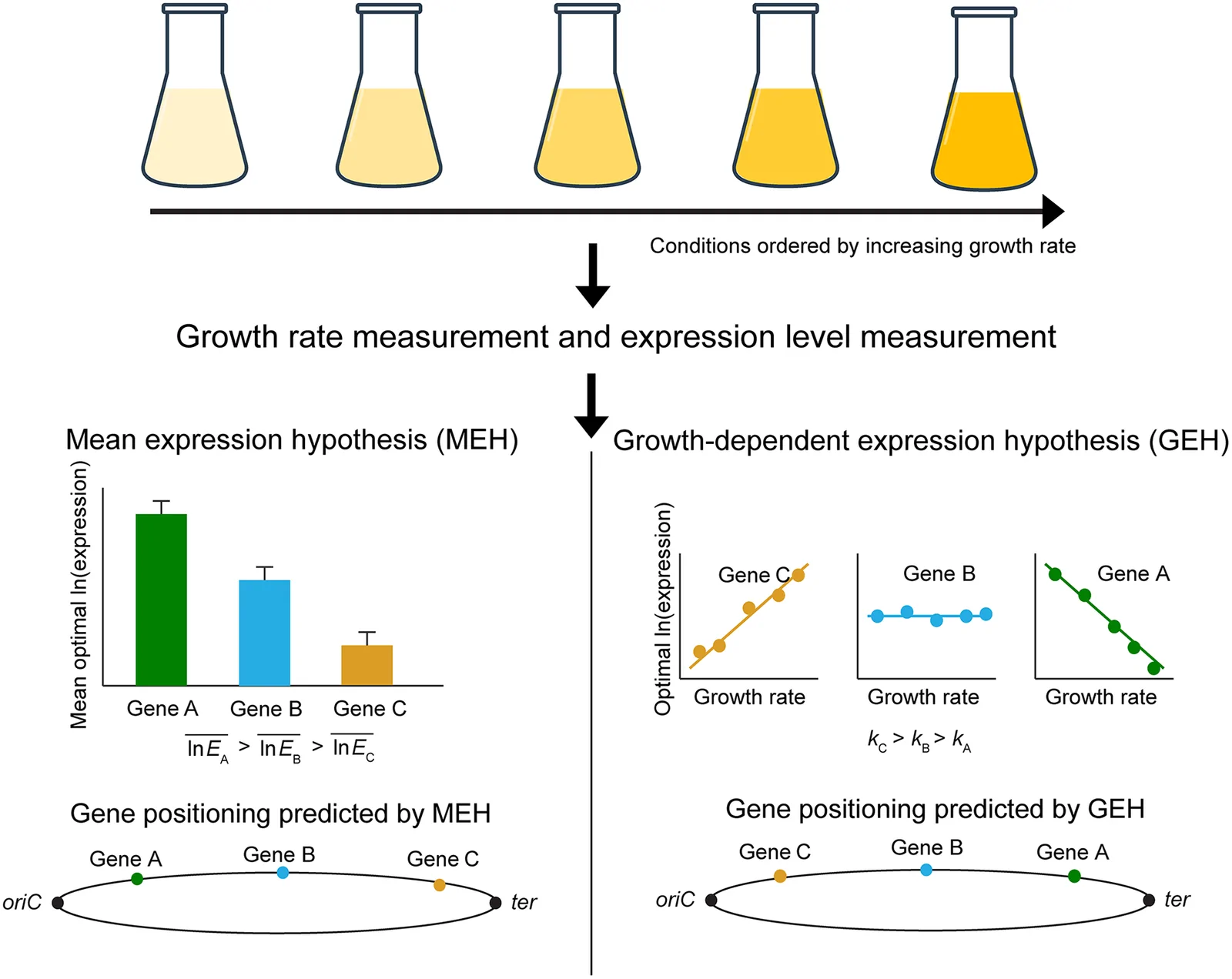
The mean expression hypothesis (MEH) and growth-dependent expression hypothesis (GEH). MEH predicts that selection favors placing genes from *oriC* to *ter* in order of decreasing mean expression ($\overset{―}{\text{ln}E}$), while GEH predicts that selection favors placing genes from *oriC* to *ter* in order of decreasing growth dependence in expression (*k*).

Because Eq (4) indicates that gene position simultaneously influences gene expression and its growth dependence, two hypotheses propose different expression components as the target of selection and make distinct predictions about gene positioning (Fig 1). The mean expression hypothesis (MEH) considers the expression level of a gene as the selection target regardless of the growth rate and predicts that selection pushes genes with higher optimal expression levels (i.e., higher $\text{ln}E_{X}$) toward *oriC* [17]. For example, in *E. coli*, *Bacillus subtilis*, and *Streptomyces*, a significant negative correlation exists between gene position *x* and expression level, with no association between gene function and *x* [21]. Under the assumption that the observed expression levels reflect optimal levels, these observations support MEH. Although MEH does not explicitly consider environmental variation, it predicts that genes with higher mean expression across environments ($\overset{―}{\text{ln}E}$) lie closer to *oriC*.

By contrast, the growth-dependent expression hypothesis (GEH) emphasizes the growth dependence in expression as a target of selection [16]. One can regress $\text{ln}\;E_{X}$ on the scaled growth rate *μ*_scaled_, which equals 0.619*μ* (see Eq 4), to obtain the expression slope *k* as a measure of the extent to which a gene’s expression is growth dependent. GEH predicts that selection pushes genes with larger positive optimal *k* toward *oriC* and genes with larger negative optimal *k* toward *ter*. An underlying assumption of GEH is that each gene has a single optimal *k* regardless of the specific environments concerned; otherwise, the *k*-based selection would vary in direction and strength depending on the environment and it would be difficult for GEH to predict gene position patterns.

Evidence supporting GEH is available from comparative genomics and experimental gene translocation. For instance, previous work reported that translation- and transcription-related genes (i.e., those encoding RNA polymerases, ribosomal RNAs, ribosomal proteins, and certain tRNAs), but not other highly expressed genes, are significantly enriched near *oriC* in hundreds of bacterial species [5]. Consistent with GEH, translocating near-*oriC* ribosomal protein genes to a position distal to *oriC* reduced cell fitness under fast-growth but not slow-growth conditions [22]. Furthermore, fitness at fast growth recovered when two copies, but not one copy, of the ribosomal protein genes were translocated to positions distal to *oriC* [22]. This latter finding, however, is consistent with both MEH and GEH. Hu et al. recently analyzed three proteomic and ribosome profiling datasets and reported higher *k* for genes with 0 < *x* < 1/3 than genes with 2/3 < *x* < 1, supporting GEH [16]. They additionally argued that MEH is unsupported because there is no clear decreasing trend from *oriC* to *ter* in the codon adaptation index of genes along the chromosome. Their finding that genes with various functions are conserved in gene position across species suggests that selection shapes gene positioning of a large number of genes, not only the translation- and transcription-related genes that were the focus of earlier studies [5,22]. However, Hu et al.’s analyses had three limitations. First, codon adaptation index does not correlate perfectly with gene expression level [23]. Second, Hu et al. did not quantitatively assess the relative support for MEH and GEH. Third, their empirical evidence for GEH is based on only three pairs of environments; the robustness and generality of their results therefore remain unknown.

Although GEH and MEH should apply to all genes in principle, it has previously been hypothesized that selection on the positioning of translation- and transcription-related genes should be especially strong [5] because of the high demand for ribosomal proteins [24] and the increasing demand for ribosomal proteins and RNA polymerases with growth rate [24,25]. As mentioned, consistent with this hypothesis, positions of translation- and transcription-related genes are biased towards *oriC* across bacterial species [5,16]. It would be interesting to further test whether these genes disproportionately drive the *oriC*-to-*ter* gradients in $\overset{―}{\text{ln}E}$ and *k*.

In the present study, we collect eight publicly available proteomics and ribosome profiling datasets in three bacterial species and a dataset of *E. coli* promoter strength measured in two environments to evaluate and compare MEH and GEH. Our analysis supports both MEH and GEH and suggests that their relative strengths vary with species. Notwithstanding, even combined, the two hypotheses have limited power explaining gene positions (*r*^2^ = 0.007 – 0.039). Our results also show that translation- and transcription-related genes disproportionately contribute to positional gradients in mean and growth-dependent expression.

## Results

### Growth rate and protein expression data

To test and compare MEH and GEH, we looked for publicly available proteomic and ribosome profiling data based on the following criteria: (1) proteomic or ribosome profiling was used to measure protein expressions, (2) the data were collected at a wide range of steady-state growth rates (a minimum of 2-fold difference between the lowest and highest growth rates), and (3) the use of wild-type strains. We did not consider mRNA expression data because natural selection on gene expression acts more directly on the protein than mRNA level [26]. We found eight datasets including the three used previously [16] (Table 1). The eight datasets employed two different experimental methods: batch cultures and accelerostat cultures where samples are taken along a continuous spectrum of dilution rates. For batch cultures, we define a unique combination of pH, temperature, and medium as a condition in a dataset. For accelerostat cultures, we define every sample as a condition in a dataset. The eight datasets included three species: *E. coli*, *B. subtilis*, and *Vibrio natriegens*.

**Table 1:** Datasets analyzed in the present study.

Dataset name	Data type	Species and strain	# of conditions	# of genes	Reference
Goelzer dataset	Proteomics	*Bacillus subtilis* BSB1	5	1002	[32]
Zhu *B. subtilis* dataset	Proteomics	*B. subtilis* 168	10	1777	[31]
Li dataset	Ribosome profiling	*Escherichia coli* MG1655	3	3742	[27]
Peebo dataset	Proteomics	*E. coli* BW25113	23	1096	[28]
Schmidt dataset	Proteomics	*E. coli* BW25113	20	2043	[29]
Valgepea dataset	Proteomics	*E. coli* MG1655	5	1203	[30]
Zhu *E. coli* dataset	Proteomics	*E. coli* NCM3722	11	1710	[31]
Zhu *V. natriegens* dataset	Proteomics	*Vibrio natriegens* ATCC 14048	11	1443	[31]

Datasets are named after the last name of the first author. Names of datasets used in [16] are underlined. Genes with non-zero expression in at least half of the conditions in each dataset are counted.

For *E. coli*, we analyzed the following five datasets, all of which used K-12 derivatives. The Li dataset included protein synthesis rate measured by ribosome profiling in three conditions where growth rate ranged from 0.74 to 1.93 h^-1^ [27]. We used the protein synthesis rate as a proxy for protein copy number, because they were found to correlate well with each other [27]. The Peebo dataset included protein concentration and growth rate data (growth rate ranging from 0.21 to 0.82 h^-1^) measured in two media in accelerostat [28]. Each medium had two runs where samples in two slightly different series of growth rates were collected. We treated each of the 23 samples from these runs as a unique condition. The Schmidt dataset included protein abundance and growth rate data (growth rate ranging from 0.12 to 1.90 h^-1^) in 20 conditions after excluding two conditions in stationary phase [29]. The Valgepea dataset included protein abundance and growth rate data (growth rate ranging from 0.11 to 0.49 h^-1^) collected at five sampling points in an accelerostat with defined minimal medium [30]. The Zhu *E. coli* dataset included protein mass fraction and growth rate data (growth rate ranging from 0.40 to 1.90 h^-1^) collected from 11 conditions [31].

For *B. subtilis*, we analyzed Zhu *B. subtilis* dataset using strain 168, and the Goelzer dataset using a derivative of strain 168, BSB1. The Zhu *B. subtilis* dataset included protein mass fraction and growth rate data (growth rate ranging from 0.44 to 2.57 h^-1^) collected from 10 conditions [31]. The Goelzer dataset included protein abundance and growth rate data (growth rate ranging from 0.3 to 1.5 h^-1^) collected from five conditions [32].

For *V. natriegens*, we analyzed the Zhu *V. natriegens* dataset using strain ATCC 14048. The dataset included protein mass fraction and growth rate data (growth rates ranging from 0.26 to 2.78 h^-1^) collected from 11 conditions [31].

### Evidence for MEH

**Fig 2:**
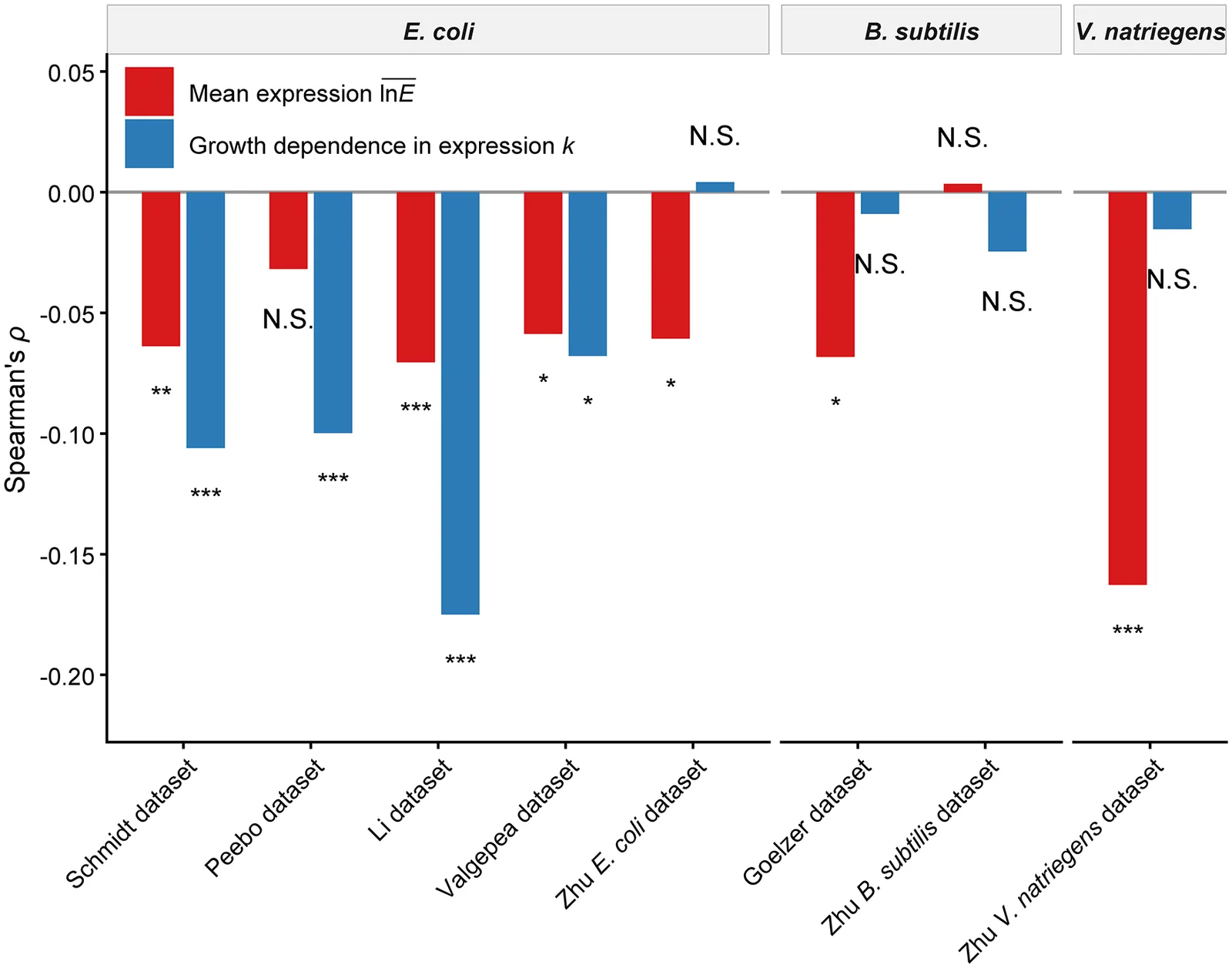
Rank correlation between gene position and mean expression ($\overset{―}{lnE};\;red$) or growth dependence in expression (*k*; blue). Gene position is normalized to a scale of [0, 1] where 0 means *oriC* and 1 means *ter*. N.S., not significant. *, *P* < 0.05. **, *P* < 0.01. ***, *P* < 0.001.

MEH predicts that genes with higher mean expression across environments are preferentially positioned near *oriC* through adaptive relocation. For a gene, we measured its mean expression by the arithmetic mean of its ln-transformed relative expression levels across all environments in a dataset, $\overset{―}{\text{ln}E}$, and assumed that the observed $\overset{―}{\text{ln}E}$ equals the optimal $\overset{―}{\text{ln}E}$. In six of the eight datasets analyzed, $\overset{―}{\text{ln}E}\;$is significantly negatively associated with *x* based on either rank (Fig 2) or linear (S1 Fig) correlation, as predicted by MEH. The remaining two datasets do not show a significant correlation (Figs 2, S1). Because all statistically significant results support MEH, we conclude that there is overall support for MEH in the datasets studied.

### Evidence for GEH

For convenience, let us refer to genes with 0 < *x* < 1/3 as *oriC* genes and those with 2/3 < *x* < 1 as *ter* genes. In support of GEH, Hu et al. reported that *oriC* genes have significantly higher expressions in faster-growing conditions than in slower-growing conditions whereas *ter* genes show the opposite trend, in three condition pairs based on two proteomic datasets and one ribosome profiling dataset [16]. Because their choice of condition pairs was arbitrary and the results may depend on this choice, we assessed robustness by including additional condition pairs from eight datasets. Specifically, we identified all growth-divergent condition pairs (absolute growth rate difference > 0.25 h^-1^ and growth rate in the faster-growing condition is more than 1.5 times that in the slower-growing condition) in each dataset. For each condition pair, we tested whether genes in a chromosomal region (*oriC* or *ter*) have significantly different expressions between the two conditions (two-sided paired Wilcoxon signed-rank test; S2 Fig). GEH predicts that *oriC* genes have higher expression in the faster-growth condition than in the slower-growth condition while the reverse is true for *ter* genes. We found that the results differ by condition pair and species. For many condition pairs in *E. coli* datasets, the observed expression differences between conditions are consistent with the GEH predictions. Nonetheless, the results are mixed in *B. subtilis* and *V. natriegens* datasets, with some condition pairs supporting GEH and others against GEH. These results are robust to the choice of growth-rate cutoffs used to define growth-divergent condition pairs (S3 and S4 Figs).

However, the above tests are not ideal because they do not explicitly compute the expression slope *k* and test GEH’s prediction of a negative correlation between *k* and *x*. To this end, we used multiple conditions in each dataset to estimate *k*. In four of the eight datasets, we found a significant, negative (rank and linear) correlation between *k* and *x* (Figs 2, S5), supporting GEH. In the remaining datasets, the rank correlation was not significant (Fig 2), although the linear correlation was significantly positive in Zhu *E. coli* dataset (S5 Fig). Because most significant results support GEH, we conclude that there is overall support for GEH in the datasets studied.

### The explanatory power of MEH and GEH

A key assumption of GEH is that each gene has a single optimal *k* across all environments. We tested this assumption by calculating the standard deviation of θ=arctan(k) and the sign consistency of θ across the five datasets of *E. coli* and across the two datasets of *B. subtilis*, respectively. We converted *k* to θ, because *k* is a slope, which can naturally be viewed as an angle ranging from -90° to 90°. We found that the sign of θ is inconsistent between the two *B. subtilis* datasets for 33% of genes and across the five *E. coli* datasets for 56% of genes (S6 Fig). The standard deviation of θ is on average 38° across the *B. subtilis* datasets and 37° across the *E. coli* datasets. Due to this high variance in θ, we compiled *E. coli* and *B. subtilis* consensus datasets from which we estimated inverse-variance weighted mean $\overset{―}{\text{ln}E}$ and *k* (see Materials and methods). Using these estimates, we assessed the relative contributions of $\overset{―}{\text{ln}E}$ and *k* in explaining the variation in gene position. Specifically, we fitted a beta regression model predicting gene position using z-score of $\overset{―}{\text{ln}E}$ and z-score of *k* (Model 1), based on the *E. coli* consensus data, *B. subtilis* consensus data, and Zhu *V. natriegens* data, respectively. MEH predicts a negative regression coefficient for $\overset{―}{\text{ln}E}$ while GEH predicts a negative regression coefficient for *k*. The hypothesis with the more negative regression coefficient contributes more to chromosomal gene positioning.

**Fig 3:**
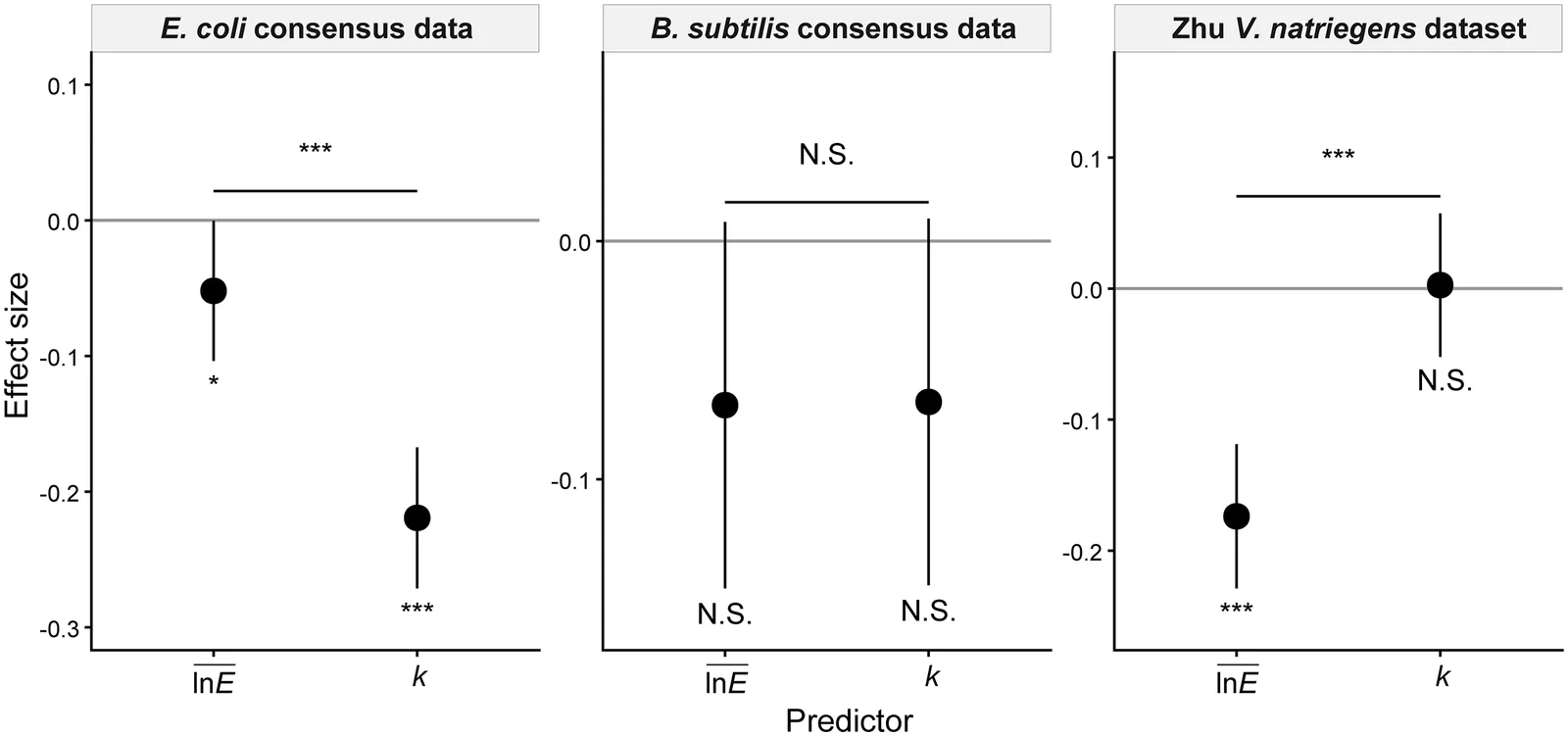
Beta regression models contrasting support for GEH and MEH. Each circle shows a regression coefficient for mean expression ($\overset{―}{\text{ln}E}$) or growth dependence in expression (*k*) in a beta regression model, with the error bar indicating its 95% confidence interval. Stars or N.S. below error bars indicate whether the regression coefficient is significantly different from 0 or not (Wald test). Stars or N.S. on brackets indicate whether the two regression coefficients in the model are significantly different or not (Wald test). N.S., not significant. *, *P* < 0.05. **, *P* < 0.01. ***, *P* < 0.001.

In the beta regression model fitted for the *E. coli* consensus data, both $\overset{―}{\text{ln}E}$ and *k* have significantly negative regression coefficients (Fig 3, S1 Table), supporting both MEH and GEH. Additionally, *k* has a significantly larger effect size than $\overset{―}{\text{ln}E}$, suggesting that GEH explains gene position better than MEH in *E. coli*. For *B. subtilis*, both $\overset{―}{\text{ln}E}$ and *k* have negative, albeit non-significant, regression coefficients. Effect sizes of $\overset{―}{\text{ln}E}$ and *k* in the model are also similar. Hence, neither GEH nor MEH is significantly supported in *B. subtilis*. For *V. natriegens*, $\overset{―}{\text{ln}E}$ has a significantly negative regression coefficient while *k* does not have a significant coefficient. The difference in effect size between $\overset{―}{\text{ln}E}$ and *k* is significant. Hence, MEH but not GEH is significantly supported in *V. natriegens*. Together, the results from the three species indicate that the relative importance of the two hypotheses varies with the species.

To estimate the total explanatory power of $\overset{―}{\text{ln}E}$ and *k* for gene position, we calculated the squared Pearson correlation coefficient (*r*^*2*^) between observed and predicted positions as a measure of goodness-of-fit for the beta regression model. We found that *r*^*2*^ ranged from 0.007 to 0.039 in the three species (S1 Table), suggesting that, even combined, $\overset{―}{\text{ln}E}$ and *k* have limited explanatory power for gene position.

### Expression adaptation through changes in gene position and promoter strength

While the above sections have demonstrated that gene position patterns are consistent with expression-related selection, a complementary question is whether the mean expression and growth-dependent expression have been optimized through changes in both gene position and promoter strength. To address this question, we analyzed previously quantified strengths of all candidate promoters in *E. coli* [33]. Specifically, Urtecho et al. sheared the *E. coli* genome into 200- to 300-nucleotide fragments with 8.5 × coverage. They then built a library where DNA fragments were placed upstream of the superfolder green fluorescent protein (sfGFP) gene including a barcode, inserted the library into a defined intergenic location in the *E. coli* genome, and performed targeted amplicon sequencing of the barcoded sfGFP transcripts to quantify the RNA levels of barcodes normalized to their DNA abundances in LB medium and M9 medium, respectively. For each nucleotide site in the genome, they calculated the median ratio of barcode RNA abundance to barcode DNA abundance across all fragments covering the site, which was considered the promoter activity of the site. They defined a candidate promoter as a genomic region where the promoter activity is continuously higher than an empirical threshold for at least 60 nucleotides. In the present study, we mapped the candidate promoters to genes they regulate and used the highest expression level within a promoter sequence as a proxy for the promoter strength (see Materials and methods).

**Fig 4:**
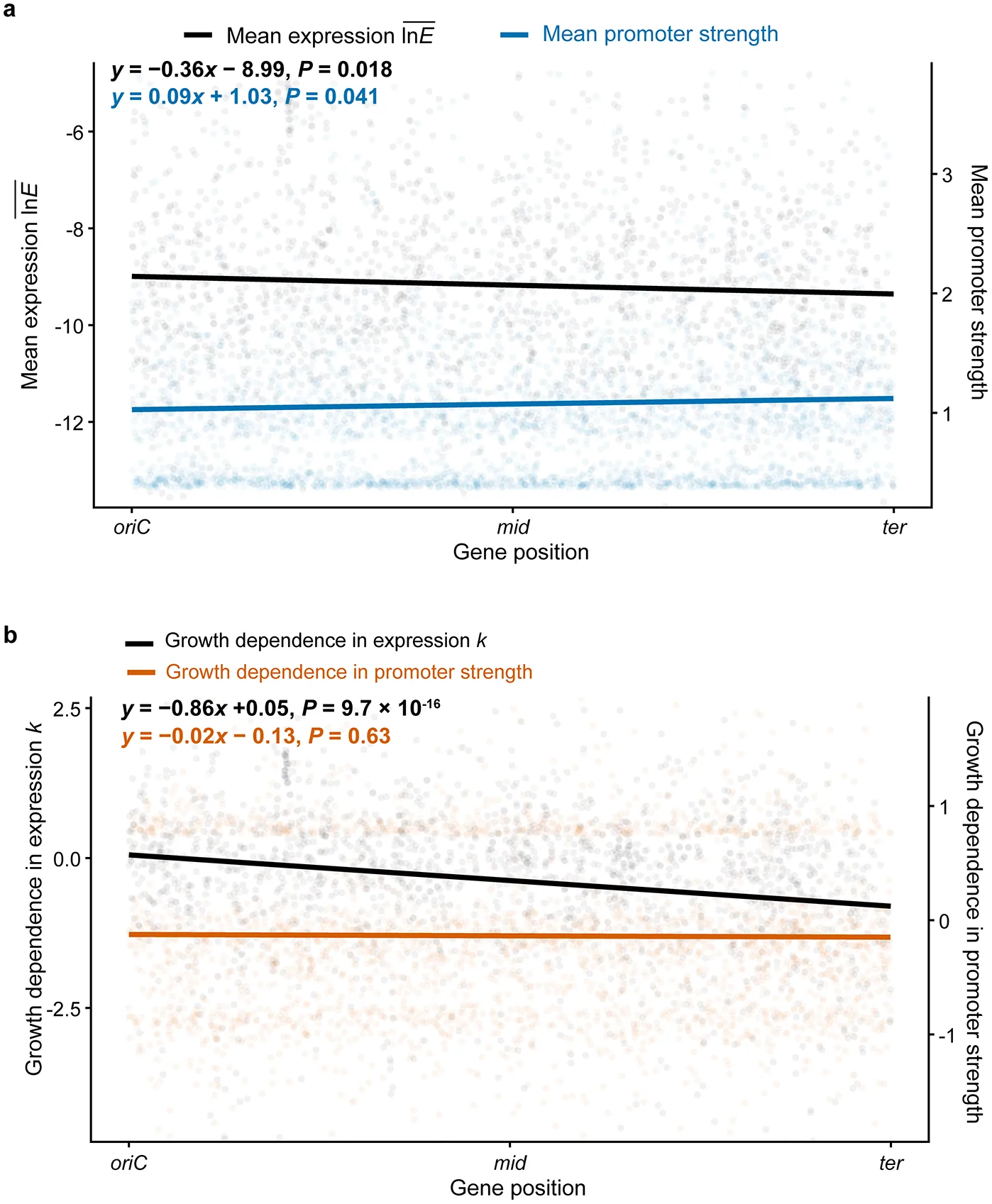
Expression and promoter strengths of *E. coli* genes. Each dot represents a gene. Expression is based on the *E. coli* consensus data. **(a)** Mean expression level (black) and mean promoter strength (blue) in LB and M9 media for each gene. **(b)** Growth dependence in expression (black) and promoter strength (orange) in LB and M9 for each gene. A line shows the linear regression of the dots of the same color as the line, with the regression equation and *P*-value of the null hypothesis of zero Pearson’s correlation coefficient also indicated in that color. Growth dependence in promoter strength is measured by the difference in ln(promoter strength) between LB and M9. For visual clarity, y-axis range for each variable is restricted to its 1st to 99th percentile but all data points are included in statistical analyses.

For each *E. coli* promoter, we computed its arithmetic mean of ln-transformed promoter strength in LB and M9 as a measure of its mean strength. We found a significant positive correlation between gene position *x* and mean promoter strength (Pearson’s *r* = 0.038, *P* = 0.041; Fig 4a). By contrast, there is a significant negative correlation between gene position *x* and observed $\overset{―}{\text{ln}E}$ in the *E. coli* consensus data (Pearson’s *r* = -0.058, *P* = 0.018; Fig 4a). The negative correlation between *x* and $\overset{―}{\text{ln}E}$ suggests that the replication-dose effect is used for mean expression optimization, whereas the positive correlation between *x* and mean promoter strength suggests that the optimization of individual promoter strengths has an overall effect of partially offsetting the replication-dose effect.

For each *E. coli* promoter, we also computed the difference in ln-transformed promoter strength between LB and M9 media as a measure of growth dependence in promoter strength. We found no significant correlation between gene position (*x*) and growth dependence in promoter strength (Pearson’s *r* = -0.009, *P* = 0.63; Fig 4b). By contrast, gene position *x* and observed *k* are significantly negatively correlated in the *E. coli* consensus data (Pearson’s *r* = -0.193, *P* = 9.7 × 10^-16^; Fig 4b). This negative correlation suggests that the growth-dependent replication-dose effect is used for optimizing growth-dependent expression. The lack of a significant correlation between gene position and growth dependence in promoter strength suggests that the optimization of the growth-dependent strengths of individual promoters likely neither reinforces nor offsets the growth-dependent replication-dose effect.

Bacterial genomes contain core and accessory genes, which are respectively shared and unshared across different genotypes of a species. Because core genes are more stably retained in genomes than accessory genes, we tested whether the relative contributions of the replication-dose effect and promoter strength to mean-expression optimization differ between core and accessory genes. Specifically, we constructed interaction models predicting mean promoter strength or $\overset{―}{\text{ln}E}$ by gene position *x*, pangenome class *C* (core genes coded as 0 and accessory genes coded as 1), and the interaction between *x* and *C* (see Materials and methods). We detected a significant positive interaction effect on $\overset{―}{\text{ln}E}$ in the *E. coli* consensus data, but no significant interaction effect on $\overset{―}{\text{ln}E}$ in the *B. subtilis* consensus data or Zhu *V. natriegens* dataset (S7 Fig). This means that the effect of gene position *x* on $\overset{―}{\text{ln}E}$ is more negative for core genes than for accessory genes in *E. coli*, supporting the conjecture of a stronger replication-dose effect in the optimization of mean expression of core genes than accessory genes. Furthermore, we detected a significant positive interaction effect on the mean promoter strength in the *E. coli* consensus data, meaning that the effect of *x* on the mean promoter strength is more positive for accessory than core genes in *E. coli*, consistent with the conjecture of a more important role of promoter strength evolution in the mean expression optimization of accessory genes than that of core genes. We fitted similar models for *k* and growth dependence in promoter strength to test whether optimization of growth dependence in expression or growth dependence in promoter strength differed for core and accessory genes in mechanism but found no significant interaction effect in any dataset (S7 Fig).

### Disproportional contribution of translation and transcription genes to positional gradients in mean and growth-dependent expression

**Fig 5:**
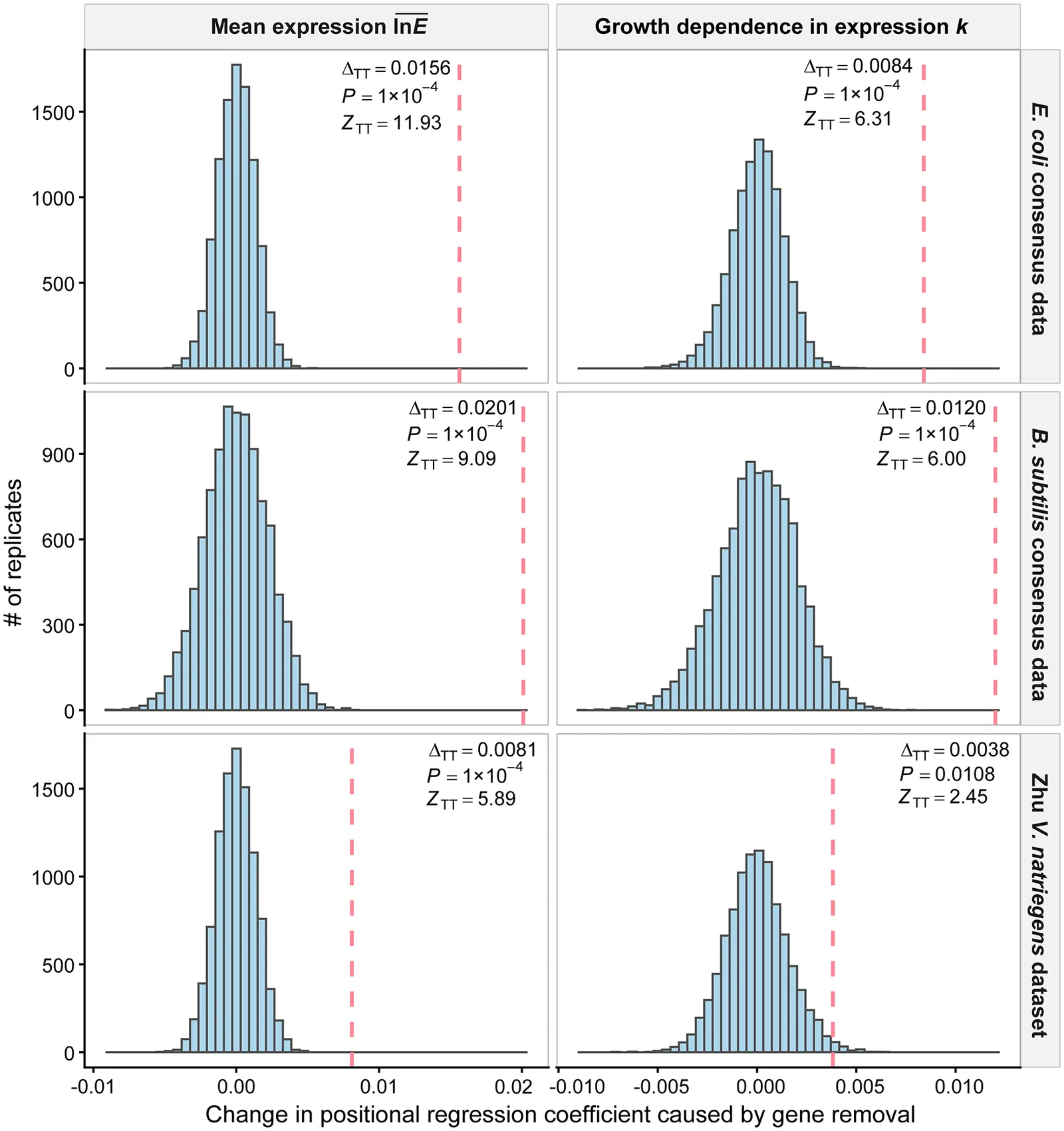
Disproportionally larger contribution of translation and transcription (TT) genes to positional gradients in mean expression ($\overset{―}{lnE}$) and growth dependence in expression (*k*). Each graph shows the distribution (blue bars) of the change in regression coefficient caused by the removal of the same number of genes as TT genes from all genes (*n* = 10,000 replicates of random gene exclusions). The red dashed line indicates $\Delta_{\text{TT}}$, the change in regression coefficient caused by the removal of TT genes from all genes. One-sided empirical *P*-value indicates the fraction of random exclusions yielding equal or greater changes in the positional regression coefficient than $\Delta_{\text{TT}}$. $Z_{\text{TT}}$ measures TT genes’ contribution to the positional gradient relative to that of the same number of random genes.

Our analyses have thus far shown an overall pattern of decreasing $\overset{―}{\text{ln}E}$ and *k* from *oriC* to *ter* across genes on the chromosome. We next tested whether these trends are similar for all genes or are especially prominent for translation and transcription (TT) genes, defined as ribosomal protein genes and RNA polymerase genes hereinafter. We first built ordinary least squares models predicting gene position *x* by $\overset{―}{\text{ln}E}$ or *k*. The regression coefficients estimate positional gradients in $\overset{―}{\text{ln}E}$ or *k*. We then examined changes in the regression coefficients when TT genes were excluded and compared the results with those when an equal number of randomly picked genes were excluded (see Materials and methods). In the *E. coli* consensus data, *B. subtilis* consensus data, and Zhu *V. natriegens* dataset, changes of regression coefficients are consistently larger by the exclusion of TT genes than by the exclusion of the same number of random genes (*P* ≤ 0.011; Fig 5), meaning that TT genes are more important than random genes in driving the positional gradients in both $\overset{―}{\text{ln}E}$ and *k*. Moreover, our results show that, relative to the contribution of random genes, the contribution of TT genes to the positional gradient is greater in the case of $\overset{―}{\text{ln}E}$ than in the case of *k* (see *Z*_TT_ in Fig 5).

Because most TT genes are core genes in the datasets analyzed, we tested whether the previously observed difference between core genes and accessory genes in the mechanism of mean expression optimization remains when TT genes are excluded. The interaction effects decrease and become non-significant for both $\overset{―}{\text{ln}E}$ and mean promoter strength when TT genes are excluded (S7 Fig). Thus, the observed difference between core genes and accessory genes in mean expression optimization is largely attributable to the enrichment of TT genes in core genes.

## Discussion

Because the position of a gene on a bacterial chromosome influences its mean expression as well as growth-dependent expression, MEH and GEH respectively posit that selection on mean and growth-dependent expression shapes gene positioning. We tested these two hypotheses using multi-environment protein expression and growth rate data and made the following observations. First, in six of the eight datasets analyzed, $\overset{―}{\text{ln}E}$ significantly negatively correlates with *x* across genes, whereas the reverse is true in none of the eight datasets. Similarly, in four of the eight datasets, *k* significantly negatively correlates with *x* across genes, whereas the reverse is true in only one of the eight datasets. Hence, both MEH and GEH are supported, although the level of support varies among species and datasets. Second, we fitted beta regression models to quantify and partition the contributions of MEH and GEH to chromosomal gene positioning. We found that *k* has a significantly larger effect than $\overset{―}{\text{ln}E}$ in *E. coli* but the reverse is true in *V. natriegens*. This difference may be biological, but it could also be caused by a difference in the number of conditions in each dataset (see below). Third, the total explanatory power of $\overset{―}{\text{ln}E}$ and *k* for gene positioning is low, with the highest *r*^2^ between predicted gene position and observed gene position being only 0.039 (in the *E. coli* consensus data). Fourth, analysis of *E. coli* promoter strengths suggests that the chromosome-wide pattern of mean expression optimization is realized by both gene relocation and compensatory promoter strength alteration, but the growth-dependent expression optimization appears to be driven by gene relocation only. Fifth, analysis after the removal of TT genes revealed that TT genes contribute to the positional gradients in $\overset{―}{\text{ln}E}$ and *k* more than other genes.

To test MEH and GEH empirically, we relied on the strong assumption that the observed $\overset{―}{\text{ln}E}$ and *k* of a gene respectively equal its optimal $\overset{―}{\text{ln}E}$ and *k*. This assumption is reasonable, because gene expression levels are predicted to be subjected to strong natural selection [34] and have been shown to become optimized relatively quickly by adaptive evolution [35]. Nonetheless, there are also findings that the observed gene expression levels of bacteria in laboratory environments are suboptimal [36,37]. If the observed expression is the sum of the optimal expression and a random noise, the correlation and regression between the observed $\overset{―}{\text{ln}E}$ (or *k*) and gene position would be weakened by the noise. In other words, the true contributions of MEH and GEH to chromosomal gene positioning may be larger than detected here. Another possibility—based on theoretical models of costs and benefits of microbial gene expression—is that a small change in gene expression for moderately or lowly expressed genes is nearly neutral [38]. Hence, a gene may have an optimal range of $\overset{―}{\text{ln}E}$ (or *k*) rather than a single optimal value of $\overset{―}{\text{ln}E}$ (or *k*). Under this scenario, a negative correlation between $\overset{―}{\text{ln}E}$ (or *k*) and gene position could arise simply from the replication-dose effect even in the absence of adaptive gene relocation. In other words, the contribution of MEH and GEH to chromosomal gene positioning could be even smaller than inferred here. Hence, it will be important to evaluate in the future the degree to which the observed gene expressions are optimal.

A key assumption of GEH is that each gene has a single optimal *k* across all environments. However, we found that the variance of *k* across datasets is large, which could have two explanations. First, measurement error of expression level and/or growth rate is large. Second, optimal *k* for the same gene varies with the environment. Consistent with the assumption made in GEH, bacterial resource allocation models with empirical data [39,40] showed a near-linear relationship between growth rate and constitutive expression (i.e., a fixed *k*) for most genes under a certain nutrient limitation regime (e.g., a range of antibiotic concentration in a certain medium). However, such linearity breaks (i.e., *k* differs among environments) when the nutrient limitation changes (e.g., a range of antibiotic concentration in different media). Because the evolution of gene position takes a long time, gene position cannot be optimized according to the *k* value in a particular environment if that environment does not last long. In changing environments, selection may have shaped gene position according to a global optimal *k*, minimizing the cumulative fitness costs arising from the mismatch between the replication-dose effect and optimal *k* in specific environments. The two potential reasons creating high variance of *k* are both mitigated in the consensus data because measurement errors tend to reduce with increasing sample size and the global optimal *k* is represented by the inverse-variance weighted *k*.

The effect size of *k* is significantly greater than that of $\overset{―}{\text{ln}E}$ in the beta regression model for *E. coli*’s consensus dataset, which includes the most conditions. However, the reverse is true in the regression model for Zhu *V. natriegens* dataset, which includes the fewest conditions. One explanation for the discrepancy is that attenuation bias, a statistical phenomenon where measurement errors of a predictor variable bias its regression coefficient towards 0, is more severe for *k* than $\overset{―}{\text{ln}E}$. That is, *k* needs to be estimated from more conditions than $\overset{―}{\text{ln}E}$ to yield an equally accurate measurement because measurement errors in growth rate induce noise in *k* but not that in $\overset{―}{\text{ln}E}$. We assessed the possibility of this statistical phenomenon using a down-sampling analysis. Consistent with the prediction of the attenuation bias, we found the regression coefficient of *k* to decline consistently as the number of conditions sampled increases in the *E. coli* consensus dataset and *B. subtilis* consensus dataset (S8 Fig). As more conditions are included in the regression model, the difference in effect size between *k* and $\overset{―}{\text{ln}E}$ becomes more negative or less positive in the *E. coli* consensus dataset and *B. subtilis* consensus dataset, suggesting that *k* may emerge as the dominant driver over $\overset{―}{\text{ln}E}$ of gene position evolution when *k* and $\overset{―}{\text{ln}E}$ are both accurately estimated in *B. subtilis*. Additionally, the observation that the beta regression model for the *B. subtilis* consensus data has poor prediction (*r* ^2^ = 0.007) and non-significant regression coefficients may also be attributable to the attenuation bias and the limited number of genes used in the analyses. Whether the same trend of a stronger attenuation bias in *k* than in $\overset{―}{\text{ln}E}$ holds for *V. natriegens* is unclear because the difference in effect size between *k* and $\overset{―}{\text{ln}E}$ remains stable regardless of the number of conditions sampled (S8 Fig). After all, we cannot exclude the possibility that the dominant driver of gene position evolution truly differs by species, given the limited number of conditions analyzed for *B. subtilis* and *V. natriegens*. In addition, whether the conclusion of this study can be generalized to other bacteria remains unknown because we analyzed only eight datasets from three species. Further studies with more datasets from phylogenetically more diverse bacterial lineages are needed to determine whether our conclusions are generalizable and whether the variation in our results among the three species reflects true differences in the biological drivers of gene positioning or an artifact of attenuation bias.

Even when combined, $\overset{―}{\text{ln}E}$ and *k* have low explanatory power for gene position. In addition to the limited strength of selection sorting gene position, another explanation is that various local regulatory processes can mask the weak global replication-dose effect [16,41]. Importantly, for mean expression, although $\overset{―}{\text{ln}E}$ correlates negatively with *x*, mean promoter strength positively correlates with *x*, indicating that the replication-dose effect on mean expression is offset by the evolution of individual promoter strengths, which may be in part because beneficial mutations relocating genes are presumably rarer than beneficial mutations altering promoter strengths. Additionally, local regulatory effects can, in some cases, be much stronger than the global replication-dose effect. For instance, it was reported that the expression of a translocated gene varied by ~300-fold depending on the gene position, but the replication-dose effect explains only a 1.4-fold expression difference between *oriC* and *ter* while the remaining variation is explained by transcriptionally silent extended protein occupancy domains, local degree of supercoiling, and neighboring gene expression [42]. Transcription-coupled DNA supercoiling produces a positive correlation in expression level between neighboring genes, which may also affect the evolution of local gene positioning on the chromosome [43]. Additionally, the poor diffusibility of mRNA molecules suggests that the location in the cell where a protein is synthesized is affected by gene position [1,44]. Thus, the spatial demand of protein can create a selection pressure on gene positioning. Furthermore, the intra-operon position and repression state were reported to shift gene expression from that predicted by the replication-dose effect [41]. Together, these local regulatory processes provide another explanation of why $\overset{―}{\text{ln}E}$ and *k* account for only a small fraction of observed variance in gene position.

The number of TT genes is at most 64 in the datasets analyzed, but TT genes have disproportionately large contributions to the positional gradients in $\overset{―}{\text{ln}E}$ and *k*. Nonetheless, even when TT genes are excluded, the negative correlation between gene position *x* and $\overset{―}{\text{ln}E}$ and that between *x* and *k* remain in several datasets, though substantially weakened (S9 Fig). This observation indicates that non-TT genes also contribute, though to a less degree, to the positional gradients. In other words, selection on gene positioning impacts both TT and non-TT genes, with comparatively larger impacts on TT genes.

For mean expression, we found that $\overset{―}{\text{ln}E}$ and promoter strength show opposite gradients from *oriC* to *ter*. In addition to the collective roles of promoter and gene relocation in optimizing gene expression, this pattern suggests that, in general, the replication-dose effect is an optional rather than an exclusive mechanism for achieving optimal mean expression. In our analysis of promoter strengths, when a promoter is detected in only one medium, we assigned 0 promoter strength to the promoter in the other medium. This can cause an underestimated mean promoter strength and an exaggerated estimate of growth dependence in promoter strength if the actual promoter strength is positive but below the detection limit. Additionally, we used the strength of the strongest promoter for a gene when multiple promoters are mapped to the gene, because transcription driven by the other promoters is thought to reflect largely molecular errors [45]. However, using the strongest promoter’s strength may not fully capture the actual promoter architecture because it was also reported that secondary transcription start sites of 26.5% of genes have at least 50% of the strengths of the corresponding major transcription start sites in *E. coli* [46]. We used the difference in ln(promoter strength) between LB and M9 media as a proxy for growth dependence in promoter strength. Ideally, we should use an integrated estimate for promoter strength synthesizing the usage of multiple promoters in various conditions and use the slope estimated from regressing ln(promoter strength) on growth rate measured in multiple growth conditions as the proxy. However, such data are currently unavailable. Due to data limitation and the less accurate proxy used in this study, the lack of a significant correlation between gene position and the difference in ln(promoter strength) between media does not exclude the possibility that growth dependence in promoter strength is involved in optimizing growth-dependent expression. Future studies utilizing integrated promoter strength data in multiple growth conditions can more powerfully test the hypothesis.

## Materials and methods

### Data acquisition and normalization

We used locus tags as the primary gene identifier to map protein abundance data to gene annotations. Locus tags are available in the datasets analyzed except for the Li dataset and Schmidt dataset. In the Li dataset, the common name of each gene is the only gene identifier available. Thus, we mapped gene common names in the Li dataset to locus tags using canonical and synonym gene names in the genome annotation (gff) file of *E. coli* MG1655. For the Schmidt dataset, we mapped UniProt accessions to locus tags using database cross-reference information in the gff file. We required each mapping to be one-to-one and used the gene common name as a fallback only when the other identifier did not yield a one-to-one mapping. We used *E. coli* MG1655 as the reference strain for all five *E. coli* datasets and *B. subtilis* 168 as the reference strain for the two *B. subtilis* datasets. Gene annotation, including gene position, *oriC* coordinate, and protein product, is based on the reference strain. Mapping the closely related strains of *E. coli* and *B. subtilis* to their reference strains is valid because the normalized gene position is highly correlated across the strains (pairwise *r* > 0.996 when ambiguous common names are excluded).

For each reference strain analyzed in this study, we acquired gene coordinates from gff files and fasta files of the genome assembly at NCBI. To find the *oriC* coordinate, we searched genome assembly ID in the DoriC database [47]. When the *oriC* coordinate was unavailable in DoriC, we searched it via Ori-Finder [48] by inputting the fasta file of the assembly. When more than one *oriC* coordinate exists in an assembly, we arbitrarily picked one coordinate, because the distance between multiple *oriC* coordinates is small (< 2000 nucleotides). Gene position *x* was then calculated by dividing the distance from a gene’s midpoint to the midpoint of the *oriC* region by one half of the chromosome length. *V. natriegens* has two chromosomes but we considered only genes on the larger chromosome because the baseline expression may be different between the two chromosomes and because approximately two-thirds of genes are on the larger chromosome. Furthermore, in the Zhu *V. natriegens* dataset, only 349 genes on the smaller chromosome, compared with 1,443 genes on the larger chromosome, have nonzero protein expression levels in at least half of the conditions.

Pangenome class data were acquired from the microbial pangenome database PanKB [49]. PanKB classified genes with prevalence ≥ 99% across all recorded strains within a species as core genes, those with prevalence ≥ 15% but < 99% as accessory genes, and those with prevalence < 15% as rare genes. In the present study, we aggregated the PanKB classes of rare genes and accessory genes into one class referred to as accessory genes.

TT genes include ribosomal protein genes and RNA polymerase genes and were identified following gene annotation in gff files. Genes encoding ribosomal proteins, ribosomal subunit proteins, alternative ribosomal proteins, or RNA degradation presenting factor (ribosomal protein S1 homolog in *B. subtilis*) were considered ribosomal protein genes. Genes encoding RNA polymerase subunits were considered RNA polymerase genes.

Proteomic and ribosome profiling data were normalized as follows. For samples from batch cultures, the expression data and growth rate data were averaged across replicates grown in the same condition. Genes with 0 read count in all conditions in a dataset were removed. If expression data were provided in the form of protein mass fraction, we converted them to protein copy fraction data with molecular weight data downloaded from UniProt [50]. If expression data were provided in the form of protein abundance, protein copy fraction was directly calculated. A pseudo-count of 10^-8^, which is on the same order of magnitude as the minimally detected copy fraction across all proteomic and ribosome profiling datasets, was added to the copy fraction of every gene in every condition. Copy fraction data were ln-transformed to obtain the expression level (lnE) used in our analysis. To reduce noise in the estimation of *k* arising from lowly expressed genes, we included only genes with nonzero expression in at least half of the conditions in each dataset. We applied this gene filter in all analyses except in the test of expression difference between faster-growing and slower-growing conditions (S2–S4 Figs) where growth dependence is defined by expression difference rather than *k*.

### Consensus data

We combined the five *E. coli* datasets and the two *B. subtilis* datasets into the *E. coli* consensus data and the *B. subtilis* consensus data, respectively. Because we analyzed only one dataset from *V. natriegens*, there was no consensus data for this species. Genes of different strains were mapped by locus tags. For the *E. coli* consensus data, we included genes that are present in at least three *E. coli* datasets. For the *B. subtilis* consensus data, we included genes that are present in both *B. subtilis* datasets. The above requirements were applied to all analyses using consensus data, except for the interaction models involving pangenome classes. The exception is intended to avoid bias towards genes with higher gene prevalence. Consensus *k* was estimated from $\frac{\sum \left(\frac{k_{i}}{{\left[SE\left(k_{i}\right)\right]}^{\text{2}}}\right)}{\sum \left(\frac{\text{1}}{{\left[SE\left(k_{i}\right)\right]}^{\text{2}}}\right)}$, where $k_{i}$ is the estimate of *k* from dataset *i* and $SE\left(k_{i}\right)$ is the standard error of $k_{i}$. Consensus $\overset{―}{\text{ln}E}$ was similarly estimated. Gene position in the consensus data is the gene position in the reference strain.

### Beta regression models

To facilitate direct comparison of effect sizes of $\overset{―}{\text{ln}E}$ and *k*, we converted the two variables to z-scores using the formula $z=\frac{y-\overset{―}{y}}{SD}$, where *y* is an original variable, $\overset{―}{y}$ is its mean, and SD is its standard deviation. We fitted the following beta regression model with function betareg() from the betareg package in R [51]:


$$\begin{array}{c} ogit(m(x_{i}))=\beta_{\text{0}}+\beta_{\text{1}}z_{\overset{―}{\text{ln}E},i}+\beta_{\text{2}}z_{k,i}\;,\;(\text{Model }\text{1}) \end{array}$$

where $x_{i}$ is the normalized chromosomal position of gene *i*, $m(x_{i})$ is the expected normalized chromosomal position of gene *i* conditional on its predictor values, $z_{\overset{―}{\text{ln}E},i}$ is the z-score of $\overset{―}{\text{ln}E}$ of gene *i*, and $z_{k,i}$ is the z-score of *k* of gene *i*. We used beta regression because the logit link in the model constrains the model-predicted mean to lie within the range of (0, 1), which is appropriate for our normalized gene position data [51].

### Promoter strength variation along the chromosome

We mapped *E. coli* candidate promoters to the genes they control, as described by the authors of the promoter strength dataset [33]. We first examined whether the nucleotide position with the highest promoter strength in a promoter overlaps with a gene; the promoter was mapped to the gene if this is the case. If no overlap was found, we mapped the promoter to the first downstream gene within 500 bp. If no downstream gene within 500 bp was found, we left the promoter unmapped. Because the same promoter may be mapped to slightly different coordinates in the data from LB and M9 media, we identified the same promoters in the two media by requiring an overlap of their coordinate range in the two media. For promoters that are present in the data from only one medium, we set their strengths to 0 in the other medium. Because the promoter strength data roughly follow a log-normal distribution, we first ln-transformed the raw promoter strength ($S_{\text{raw}}$) to $S_{\text{transformed}}=\text{ln}(S_{\text{raw}}+\text{1})$. We then calculated the arithmetic mean of $S_{\text{transformed}}$ in the two media for each promoter. We kept the promoter with the highest mean promoter strength for each gene with multiple promoters. The difference in $S_{\text{transformed}}$ between LB and M9 for a promoter was used as a measure of its growth dependence in strength.

### Pangenome class interaction models

To test whether the relative contributions of the replication-dose effect and promoter strength to the optimization of mean expression or that of growth-dependent expression differ between core genes and accessory genes, we examined the interaction effect between gene position *x* and pangenome class *C* in the regression model:


$$\begin{array}{c} Y_{i}=\beta_{\text{0}}+\beta_{\text{1}}x_{i}+\beta_{\text{2}}C_{i}+\beta_{\text{3}}\left(x_{i}\times C_{i}\right)+\varepsilon_{i}\;,\;(\text{Model }\text{2}) \end{array}$$

where $Y_{i}$ is $\overset{―}{\text{ln}E}$, *k*, mean promoter strength, or growth dependence in promoter strength of gene *i*, $x_{i}$ is the chromosomal position of gene *i*, $C_{i}$ is pangenome class of gene *i*, $x_{i}\times C_{i}$ = $x_{i}C_{i}$ is the interaction between pangenome class and position of gene *i*, and $\varepsilon_{i}$ is the error term for gene *i*. $C_{i}$ = 0 for core genes and 1 for accessory genes. $\beta_{\text{3}}$ is thus the difference between the effect of gene position on $\overset{―}{\text{ln}E}$, *k*, mean promoter strength, or growth dependence in promoter strength for accessory genes and that for core genes. *P*-value was determined by a *t*-test examining whether $\beta_{\text{3}}$ is significantly different from 0.

### Removal of translation and transcription (TT) genes

To test whether TT genes contribute disproportionally to the positional gradients in $\overset{―}{\text{ln}E}$ and *k*, we examined the attenuation of the effects when TT genes are excluded and compared it with that when the same number of randomly picked genes are removed. Let us denote the regression coefficients in the ordinary least squares models predicting gene position *x* by z-scored $\overset{―}{\text{ln}E}$ (or z-scored *k*) as $\beta_{\text{all}}$, $\beta_{\text{all}-\text{TT}}$, and $\beta_{\text{all}-\text{random}}$ when all genes, all genes except TT genes, and all genes except the same number of randomly picked genes as TT genes are fitted to the model, respectively. We drew the set of random genes 10,000 times to obtain a distribution of $\beta_{\text{all}-\text{random}}$. The change in regression coefficient caused by excluding TT genes is $\Delta_{\text{TT}}=\beta_{\text{all}-\text{TT}}{-\beta }_{\text{all}}$ while the change in regression coefficient caused by excluding random genes is $\Delta_{\text{random}}=\beta_{\text{all}-\text{random}}{-\beta }_{\text{all}}$. A typical positional gradient—decreasing $\overset{―}{\text{ln}E}$ or *k* from *oriC* to *ter*—produces a negative β. If TT genes indeed contribute more than random genes to the typical positional gradient, we expect $\Delta_{\text{TT}}$ to be positive. We then quantified the disproportional contribution of TT genes to the positional gradient by $Z_{\text{TT}}=\frac{\Delta_{\text{TT}}\;-\;mean(\Delta_{\text{random}})}{SD(\Delta_{\text{random}})}$, where $SD(\Delta_{\text{random}})$ is the standard deviation of $\Delta_{\text{random}}$. One-sided empirical *P*-value was defined as the fraction of random gene exclusion replicates that resulted in ${\Delta_{\text{random}}\geq \Delta }_{\text{TT}}$, with one added to both the numerator and the denominator of the fraction.

### Down-sampling

To assess whether the difference in modeling from different datasets was caused by attenuation bias, we fitted beta regression models with down-sampled conditions in each species. For *E. coli*, we included genes that are present in at least three individual *E. coli* datasets. For *B. subtilis*, we included genes that are present in at least two individual *B. subtilis* datasets. We first pooled all conditions across datasets within a species into a global condition pool while keeping a tag for each condition to record the source dataset. Let *N* represent the total number of conditions in the global pool. We evaluated subsample size *n* ranging from 5 to *N* in step size of *s* (*s* = 5 for *E. coli* and 1 for *B. subtilis* and *V. natriegens*). For each subsample size *n*, we ran 100 independent replicates. In each replicate, we randomly sampled *n* conditions without replacement from the global condition pool. We first calculated $\overset{―}{\text{ln}E}$ and *k* for each individual source dataset and then calculated consensus $\overset{―}{\text{ln}E}$ and *k* for *E. coli* and *B. subtilis*. Z-scores of *k* and $\overset{―}{\text{ln}E}$ were fitted to the beta regression model (Model 1). A source dataset was omitted when fewer than three conditions from that dataset were sampled in a replicate. A replicate was skipped when all source datasets were omitted. This led to 55 usable replicates for *n* = 5 for *E. coli* and 100 usable replicates in all other subsampling. The mean regression coefficients across all usable replicate models were plotted in S8 Fig to show changes of regression coefficients of *k* and $\overset{―}{\text{ln}E}$ with the number of conditions sampled.

### Statistical software and visualization

All statistical analyses were performed in R (version 4.4.2). Figures were generated using R package ggplot2 [52].

### Data and code availability

Relevant data and code can be obtained from https://github.com/ruiqiy/Bacterial-gene-positioning/tree/main/Revision_1.

## Supporting information

S1 Table Beta regression model results. (PDF)

S1 FigMean expression ($\overset{―}{lnE}$) of each gene as a function of its chromosomal position.(PDF)

S2 Fig Median expression fold-difference between faster- and slower-growing conditions for *oriC* genes or *ter* genes. (PDF)

S3 Fig Same as S2 Fig except that the growth rate difference criterion is changed to absolute growth rate difference > 0.1 h^-1^ and the growth rate in the faster-growth condition is more than 1.2 times that in the slower-growth condition. (PDF)

S4 Fig Same as S2 Fig except that all condition pairs with different growth rates within a dataset are included. (PDF)

S5 Fig Growth dependence in expression (*k*) of each gene as a function of its chromosomal position. (PDF)

S6 FigDistribution of the standard deviation of the angle of growth dependence in expression (θ) from the two *B. subtilis* datasets or five *E. coli* datasets.(PDF)

S7 Fig Regression coefficient of the interaction effect between gene position *x* and pangenome class (core genes coded as 0 and accessory genes coded as 1). (PDF)

S8 FigChanges in regression coefficients of *k* and $\overset{―}{lnE}$ in beta regression models as the number of conditions sampled increases.(PDF)

S9 FigRank correlation between gene position and mean expression ($\overset{―}{lnE}$; red) or growth dependence in expression (*k*; blue) across all genes excluding TT genes.(PDF)
