# Supplementary material for: Bacterial chromosomal gene positioning is likely shaped by selection on both mean and growth-dependent expression
Source: PLoS Genet. 2026 Sep 21;22(9):e1012316. doi: 10.1371/journal.pgen.1012316 (PMC13607872; doi:10.1371/journal.pgen.1012316)
Supplement: Supplementary file 1 [file pgen.1012316.s001.pdf]

S1 Table. Beta regression model results

Dataset	# of genes	Predictor	Coefficient ± SE	*P*	*P* (diff)	r^2	VIF
E. coli consensus	1700	*k*	-0.219 ± 0.027	1.3×10^{-16}	1.29×10^{-5}	0.039	1.003
		$\overline{\ln E}$	-0.052 ± 0.026	0.048			
B. subtilis consensus	951	*k*	-0.068 ± 0.039	0.085	0.98	0.007	1.227
		$\overline{\ln E}$	-0.069 ± 0.039	0.079			
Zhu *V. natriegens*	1443	*k*	0.003 ± 0.028	0.92	3.0×10^{-6}	0.031	1.008
		$\overline{\ln E}$	-0.174 ± 0.028	5.8×10^{-10}			

# of genes: number of genes used in regression models.
Coefficient: regression coefficient estimate.
SE: standard error of the regression coefficient estimate.
P: *p*-value of the null hypothesis that the regression coefficient = 0.
P (diff): *p*-value of the null hypothesis that the two regression coefficients are equal (Wald test).
r^2: squared Pearson's *r* between observed gene position and predicted gene position.
VIF: variance inflation factor, showing minimal collinearity between the two predictors in each species.
