# Supplementary material for: Bacterial chromosomal gene positioning is likely shaped by selection on both mean and growth-dependent expression
Source: PLoS Genet. 2026 Sep 21;22(9):e1012316. doi: 10.1371/journal.pgen.1012316 (PMC13607872; doi:10.1371/journal.pgen.1012316)
Supplement: Supplementary file 2 [file pgen.1012316.s002.pdf]

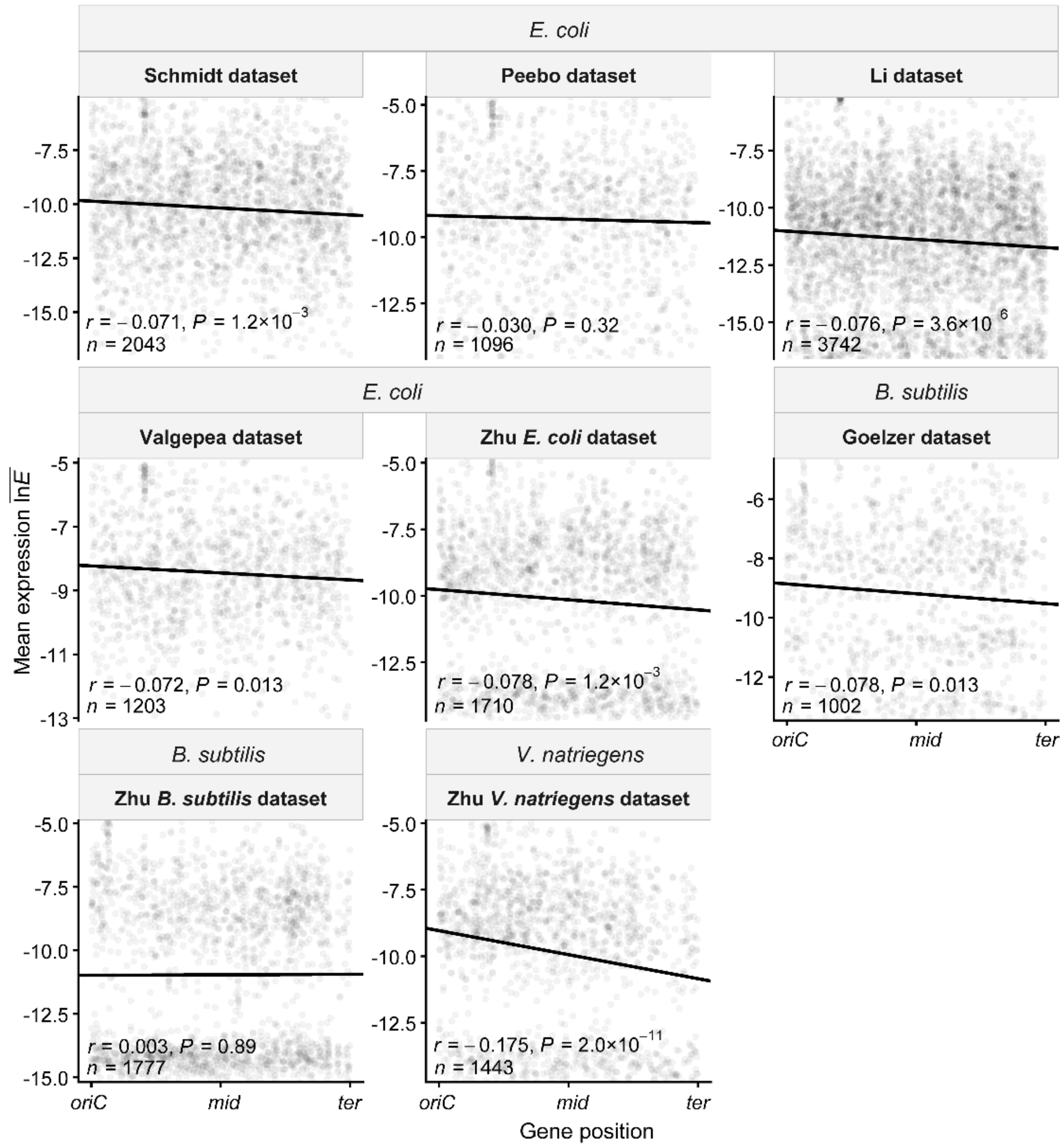

S1 Fig. Mean expression ($\overline{\ln E}$) of each gene as a function of its chromosomal position. Each dot represents a gene. Solid lines are regression lines fitted to ordinary least squares models. Pearson's correlation coefficient (r) and its P-value are shown for each dataset. For visual clarity, the y-axis range is restricted to the 1st to 99th percentiles of $\overline{\ln E}$ but all data points are included in the regression models.
