# Supplementary material for: Bacterial chromosomal gene positioning is likely shaped by selection on both mean and growth-dependent expression
Source: PLoS Genet. 2026 Sep 21;22(9):e1012316. doi: 10.1371/journal.pgen.1012316 (PMC13607872; doi:10.1371/journal.pgen.1012316)
Supplement: Supplementary file 3 [file pgen.1012316.s003.pdf]

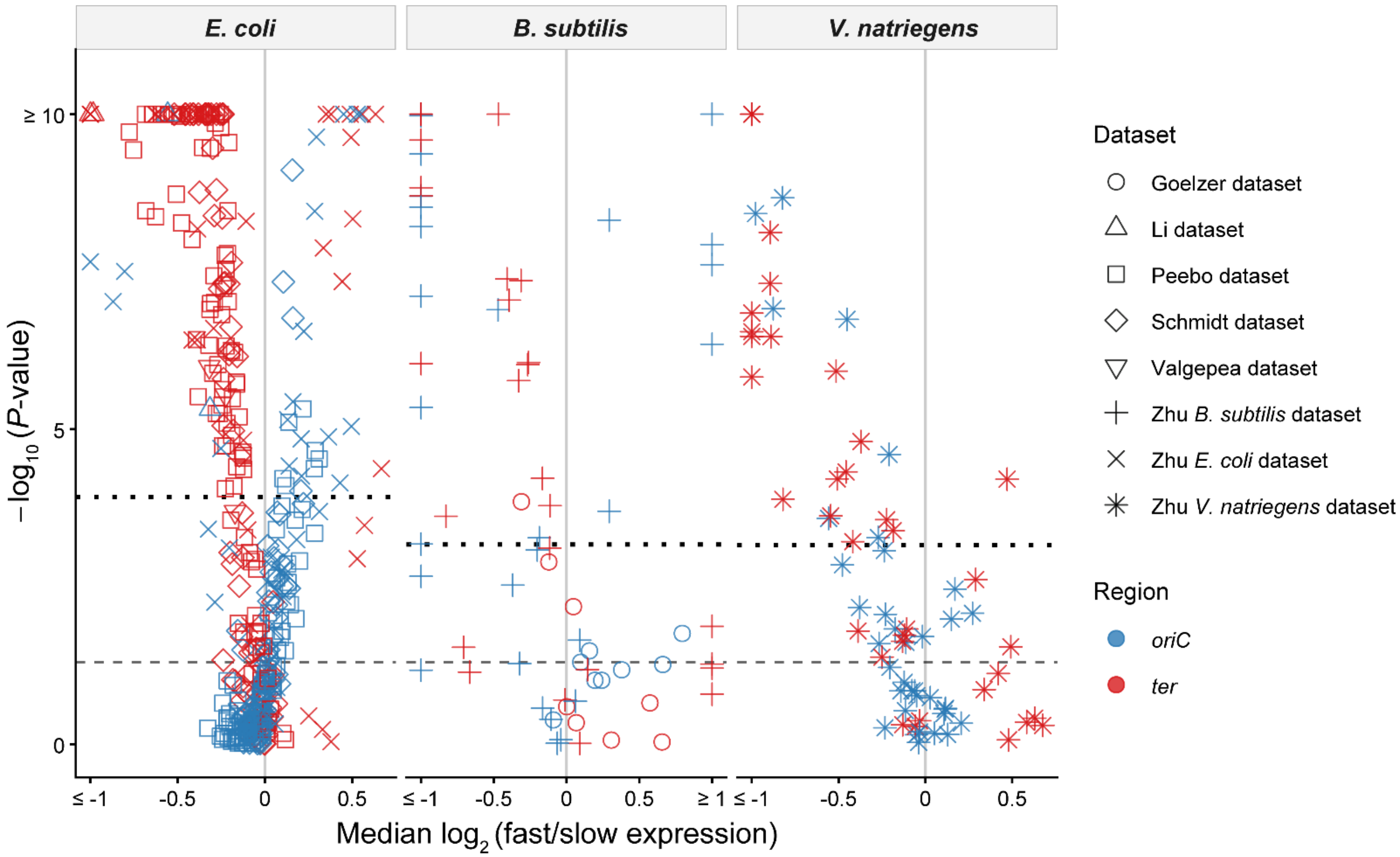

S2 Fig. Median expression fold-difference between faster- and slower-growing conditions for *oriC* genes or *ter* genes. Each dot represents all genes in a chromosomal region (*oriC* or *ter*) for a comparison between a fast- and a slow-growth condition within a dataset. All condition pairs satisfying the growth rate difference criterion used (absolute growth rate difference > 0.25 h^{-1} and the growth rate in the faster-growth condition is more than 1.5 times that in the slower-growth condition) within a dataset are included. Genes with nonzero expression in at least one condition within a condition pair are included. *P*-value is calculated using a paired two-sided Wilcoxon test comparing the expression of *oriC* genes (or *ter* genes) between the two conditions within a condition pair. GEH predicts that *oriC* genes (blue dots) are enriched on the positive side of the x-axis while *ter* genes (red dots) are enriched on the negative side of the x-axis. Dashed lines indicate canonical $P = 0.05$. Dotted lines indicate $q = 0.05$ where q is Bonferroni-corrected P.
