# Supplementary material for: Bacterial chromosomal gene positioning is likely shaped by selection on both mean and growth-dependent expression
Source: PLoS Genet. 2026 Sep 21;22(9):e1012316. doi: 10.1371/journal.pgen.1012316 (PMC13607872; doi:10.1371/journal.pgen.1012316)
Supplement: Supplementary file 4 [file pgen.1012316.s004.pdf]

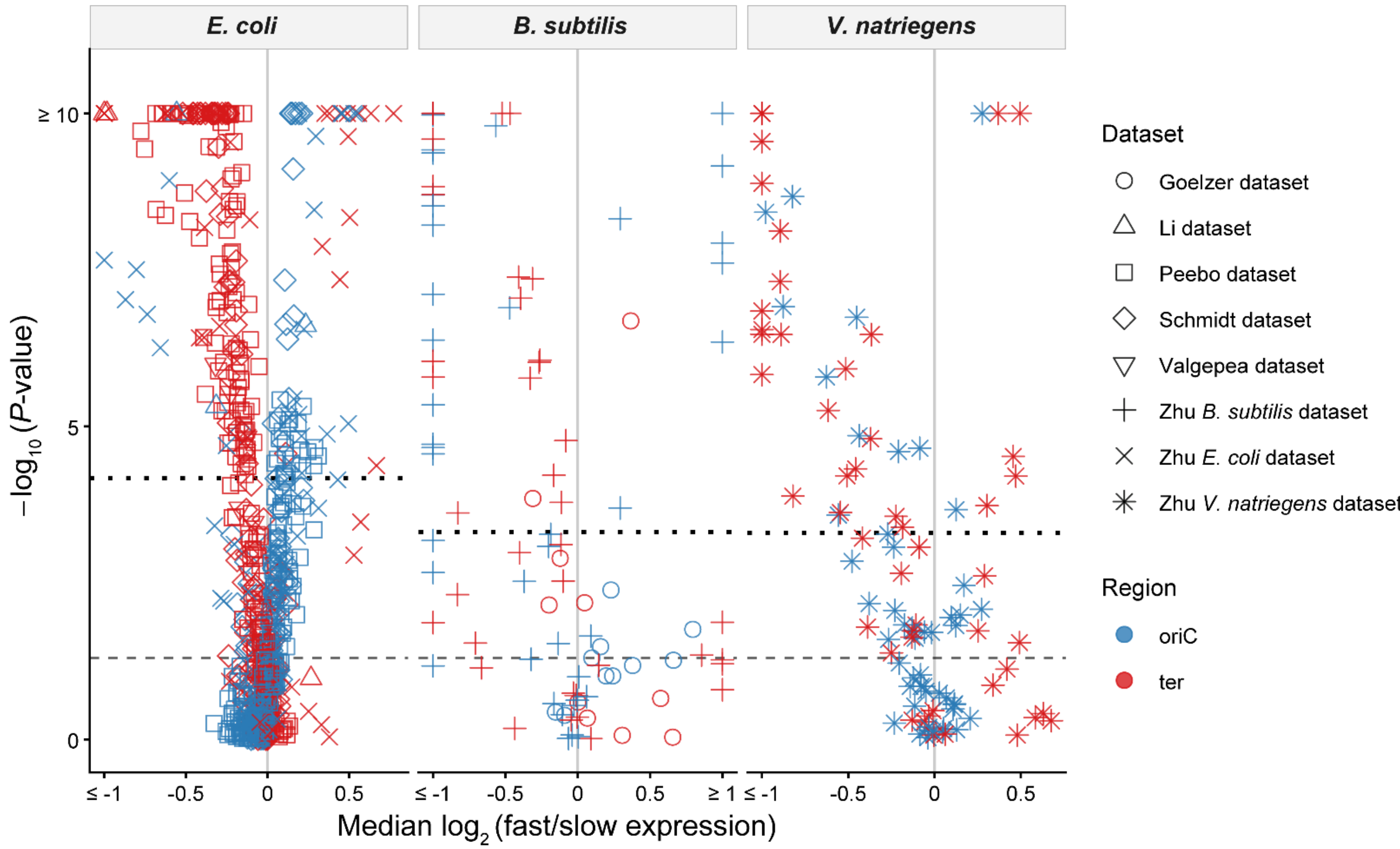

S3 Fig. Same as S2 Fig except that the growth rate difference criterion is changed to absolute growth rate difference > 0.1 h^{-1} and the growth rate in the faster-growth condition is more than 1.2 times that in the slower-growth condition.
