# Supplementary material for: Bacterial chromosomal gene positioning is likely shaped by selection on both mean and growth-dependent expression
Source: PLoS Genet. 2026 Sep 21;22(9):e1012316. doi: 10.1371/journal.pgen.1012316 (PMC13607872; doi:10.1371/journal.pgen.1012316)
Supplement: Supplementary file 5 [file pgen.1012316.s005.pdf]

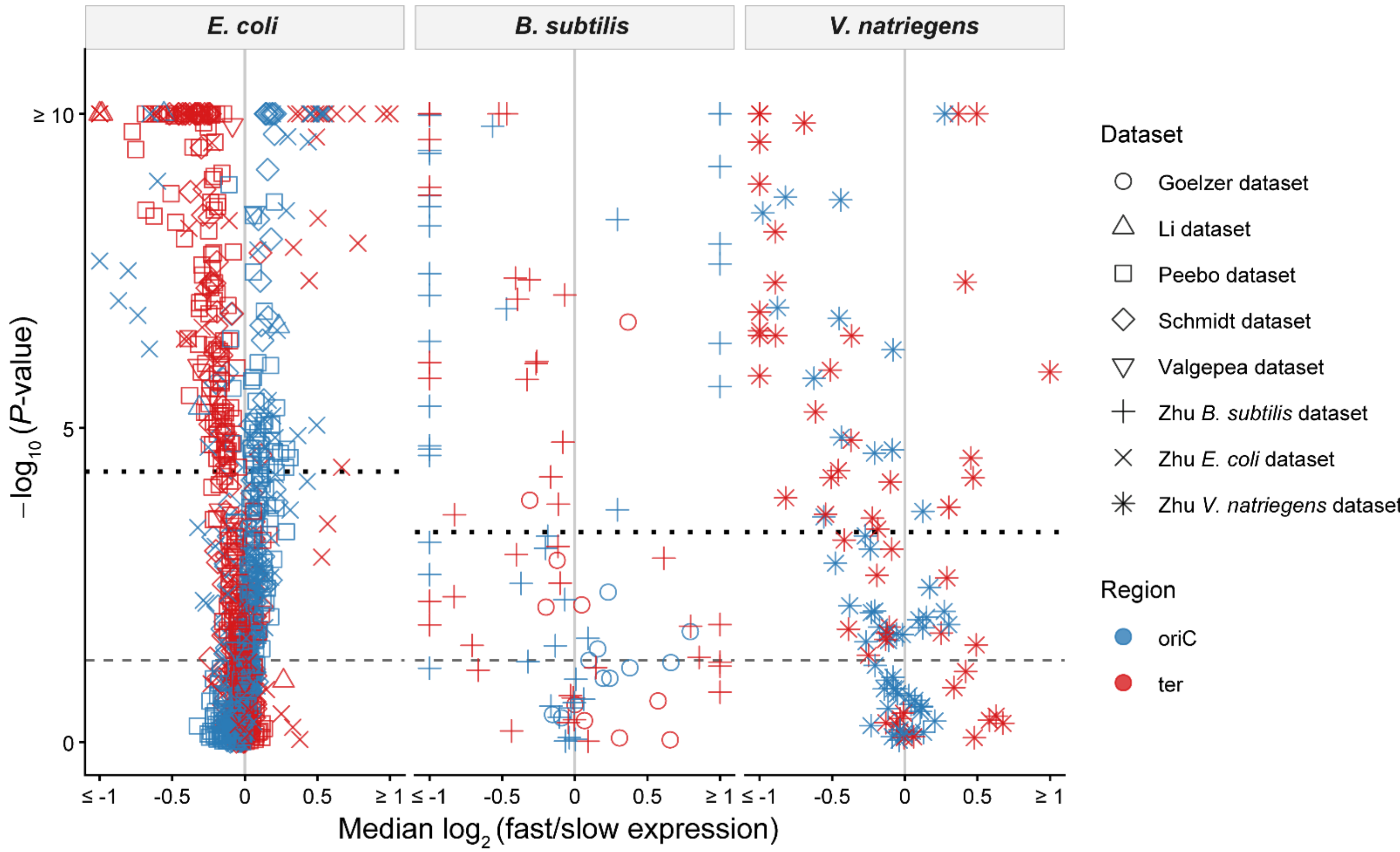

S4 Fig. Same as S2 Fig except that all condition pairs with different growth rates within a dataset are included.
