# Supplementary material for: Bacterial chromosomal gene positioning is likely shaped by selection on both mean and growth-dependent expression
Source: PLoS Genet. 2026 Sep 21;22(9):e1012316. doi: 10.1371/journal.pgen.1012316 (PMC13607872; doi:10.1371/journal.pgen.1012316)
Supplement: Supplementary file 7 [file pgen.1012316.s007.pdf]

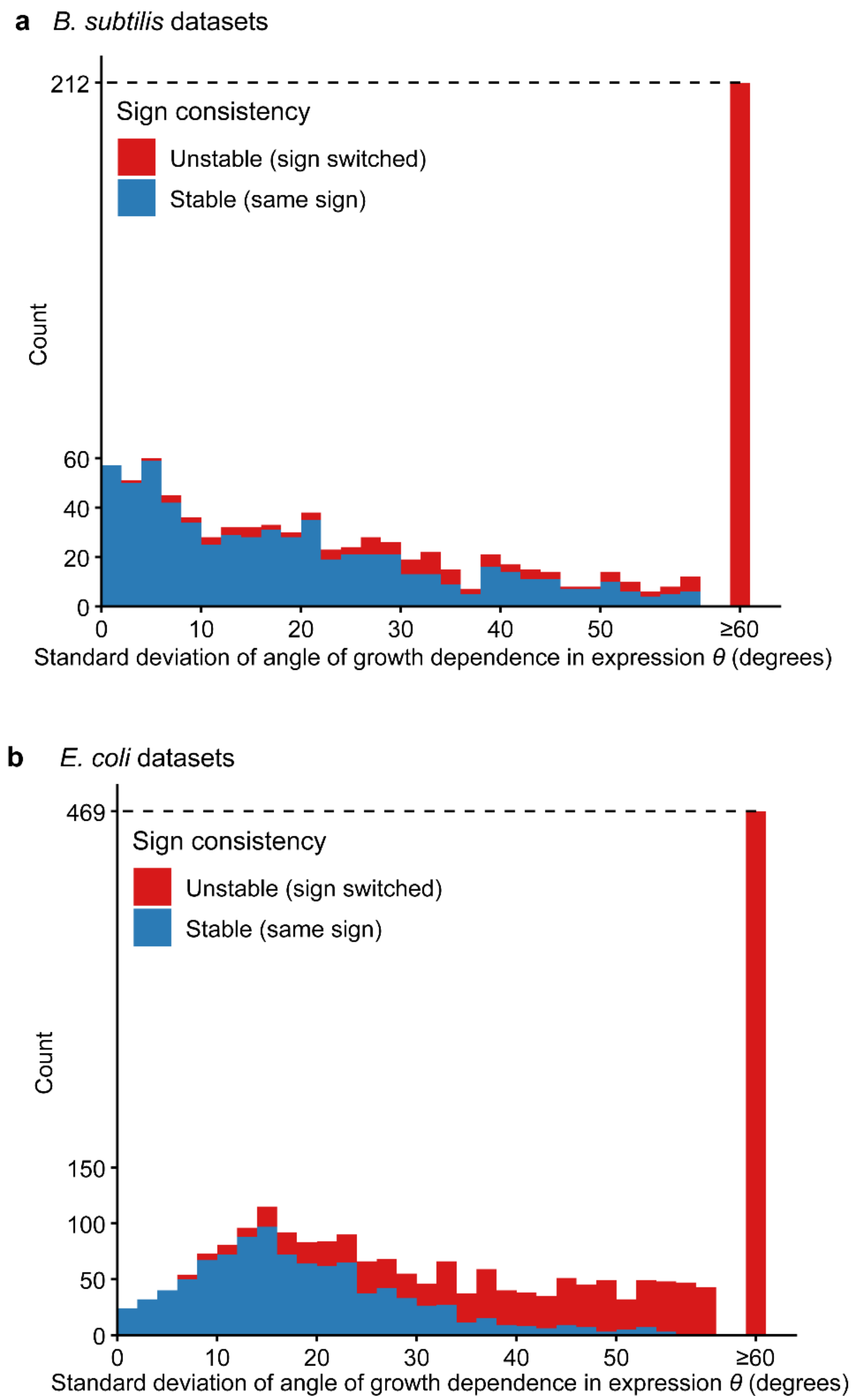

S6 Fig. Distribution of the standard deviation of the angle of growth dependence in expression (θ) from the two *B. subtilis* datasets (**a**) or five *E. coli* datasets (**b**). The height of each bar shows the number of genes in a bin of standard deviation. Each bar is further divided into two categories of genes based on their sign consistency across datasets: unstable genes (sign switched across datasets, shown in red) and stable genes (sign unchanged across available datasets, shown in blue). All genes with a standard deviation of θ of 60° or greater are aggregated in the last bin (≥60).
