# Supplementary material for: Bacterial chromosomal gene positioning is likely shaped by selection on both mean and growth-dependent expression
Source: PLoS Genet. 2026 Sep 21;22(9):e1012316. doi: 10.1371/journal.pgen.1012316 (PMC13607872; doi:10.1371/journal.pgen.1012316)
Supplement: Supplementary file 8 [file pgen.1012316.s008.pdf]

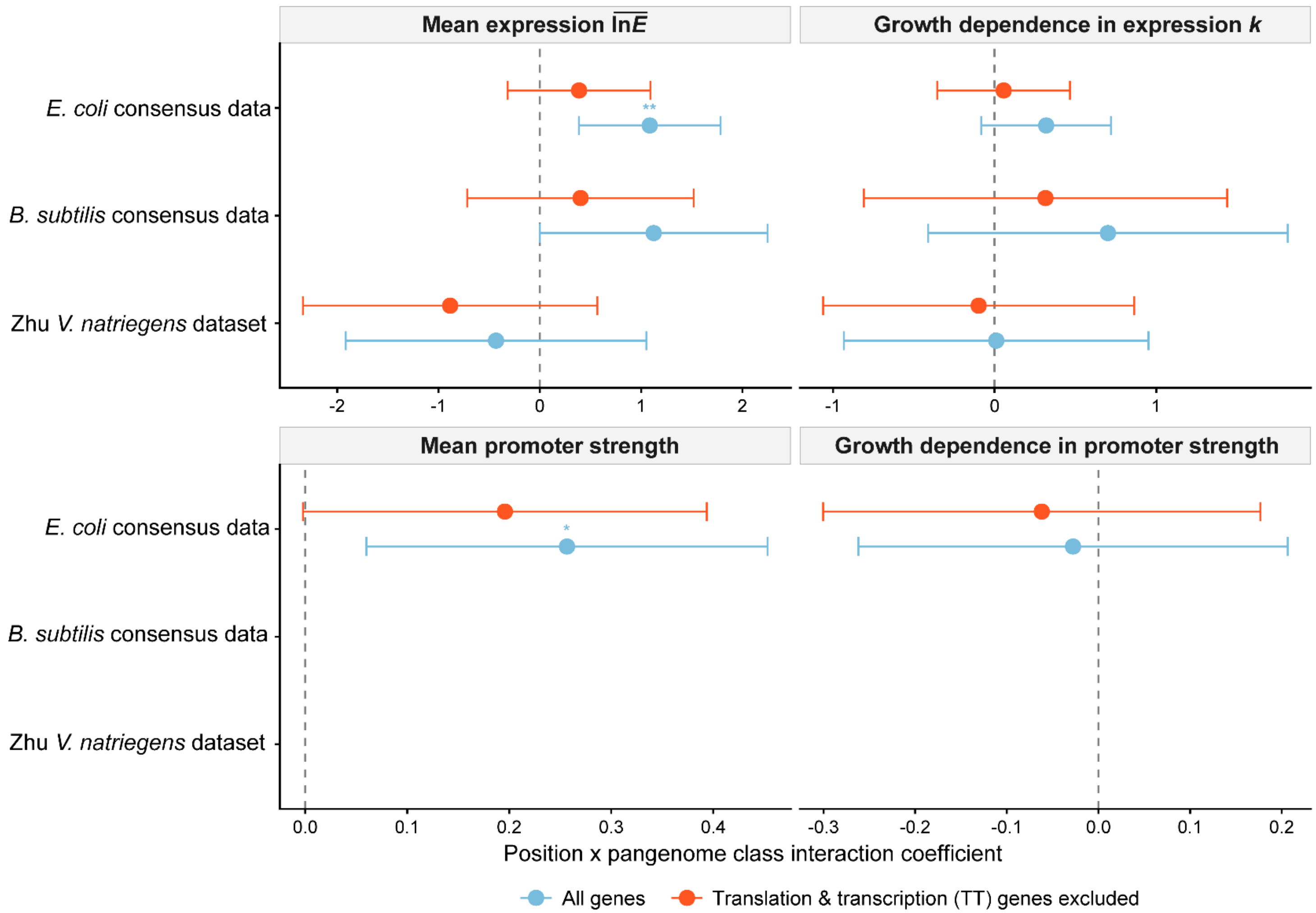

S7 Fig. Regression coefficient of the interaction effect between gene position *x* and pangenome class (core genes coded as 0 and accessory genes coded as 1). Error bars show 95% confidence intervals. *P*-value is determined by a *t*-test examining whether the regression coefficient is significantly different from 0. *, $P < 0.05$. **, $P < 0.01$.
