# Supplementary material for: Bacterial chromosomal gene positioning is likely shaped by selection on both mean and growth-dependent expression
Source: PLoS Genet. 2026 Sep 21;22(9):e1012316. doi: 10.1371/journal.pgen.1012316 (PMC13607872; doi:10.1371/journal.pgen.1012316)
Supplement: Supplementary file 9 [file pgen.1012316.s009.pdf]

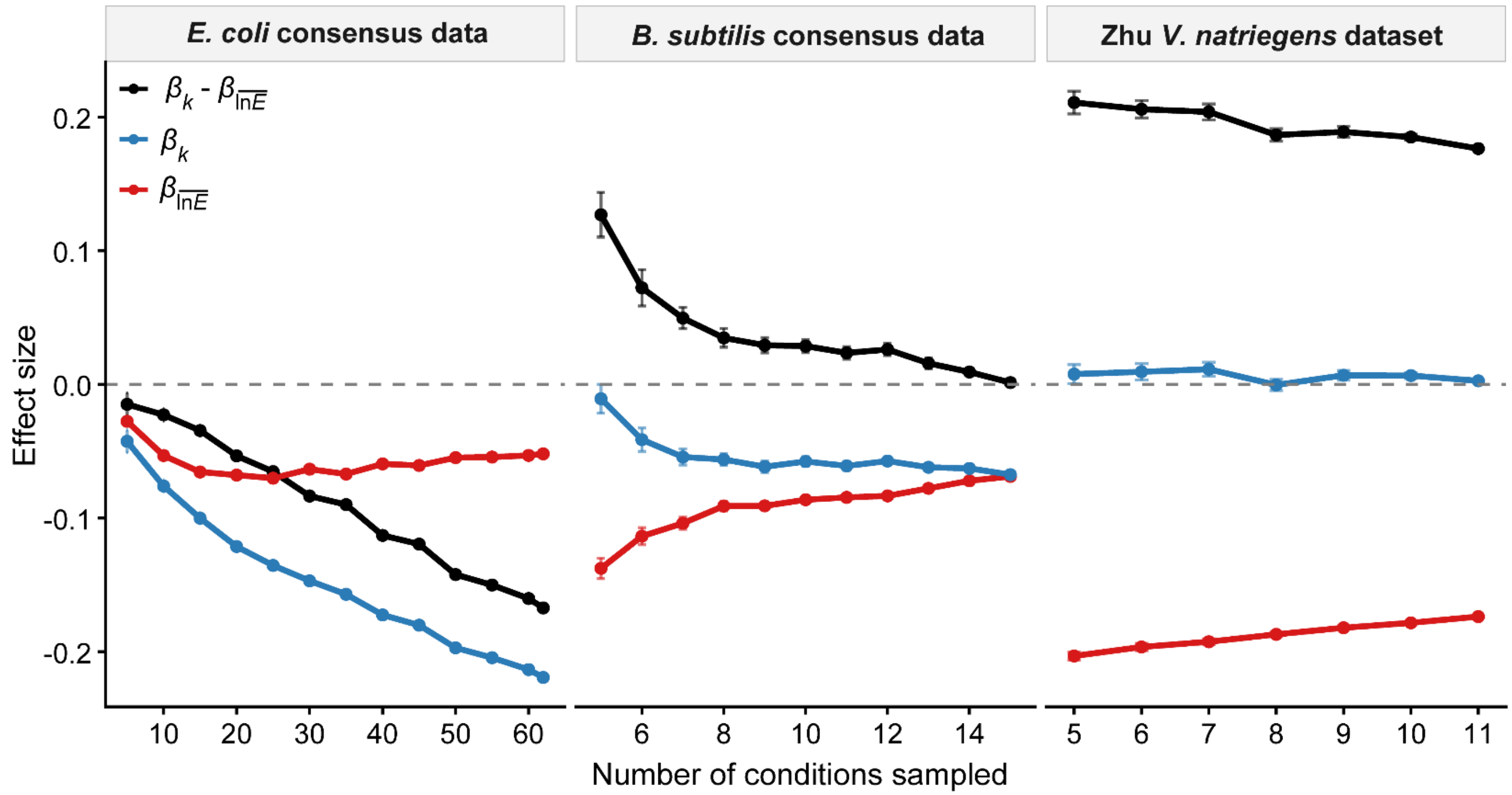

S8 Fig. Changes in regression coefficients of k and $\overline{\ln E}$ in beta regression models as the number of conditions sampled increases. For each sample size, 100 condition sets were randomly sampled to fit a beta regression model predicting gene position by z-scored k and $\overline{\ln E}$. A source dataset was omitted when fewer than three conditions from that dataset were sampled in a replicate. A replicate was skipped when no source dataset contributed at least three conditions. This led to 55 usable replicates for n = 5 for *E. coli* and 100 usable replicates in all other subsampling. Each dot shows the mean regression coefficient (or the mean difference between regression coefficients) across usable replicates. Error bars represent the standard error of the mean. Blue lines indicate the regression coefficients of k (β_k). Red lines indicate the regression coefficients of $\overline{\ln E}$ ($\beta_{\overline{\ln E}}$). Black lines indicate the difference between β_k and $\beta_{\overline{\ln E}}$.
