# Supplementary material for: Bacterial chromosomal gene positioning is likely shaped by selection on both mean and growth-dependent expression
Source: PLoS Genet. 2026 Sep 21;22(9):e1012316. doi: 10.1371/journal.pgen.1012316 (PMC13607872; doi:10.1371/journal.pgen.1012316)
Supplement: Supplementary file 10 [file pgen.1012316.s010.pdf]

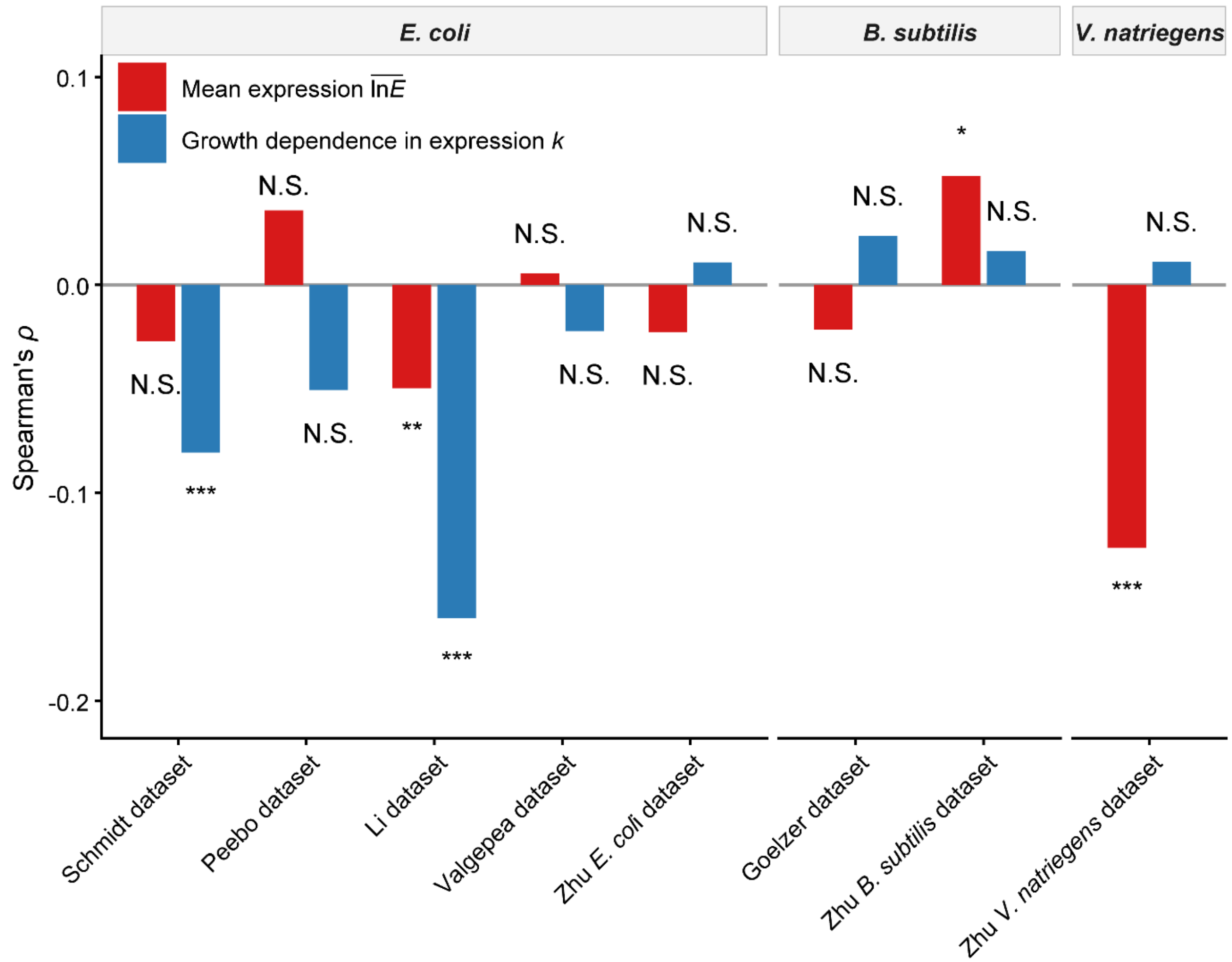

S9 Fig. Rank correlation between gene position and mean expression ($\overline{\ln E}$; red) or growth dependence in expression (k; blue) across all genes excluding TT genes. N.S., not significant. *, $P < 0.05$. **, $P < 0.01$. ***, $P < 0.001$.
