# Supplementary material for: Bacterial chromosomal gene positioning is likely shaped by selection on both mean and growth-dependent expression
Source: PLoS Genet. 2026 Sep 21;22(9):e1012316. doi: 10.1371/journal.pgen.1012316 (PMC13607872; doi:10.1371/journal.pgen.1012316)
Supplement: Supplementary file 11 [file pgen.1012316.s012.pdf]

This manuscript examines whether bacterial chromosomal gene positioning is shaped by selection on mean expression (MEH) and/or growth-dependent expression (GEH). To do this, the study examines eight multi-condition proteomic/ribosome-profiling datasets from three bacterial species, along with an *E. coli* promoter dataset, the authors report that both mean expression and expression slope with growth rate tend to decrease from oriC to ter. They conclude that both MEH and GEH contribute to gene positioning, although with modest explanatory power.

The manuscript addresses an interesting and important question in bacterial genome organization. The conceptual framing is clear, the compilation of multiple public datasets is a strength, and the conclusion that any effect is modest is appropriately cautious. The promoter-strength analysis is also potentially valuable.

That said, I have several substantive concerns that should be addressed before publication.

Major:

1. The manuscript should more clearly distinguish that the findings are consistent with adaptive positioning rather than providing direct evidence that selection shaped positioning.
2. With such a small sample size of transcriptomic data where only ~half had significant results, it isn't appropriate to assume results are globally applicable. A call to apply this analysis to more datasets from more varied bacterial species to better understand trends should be included in the discussion. For example, statements such as "clear evidence for MEH/GEH" feel too strong considering the modest correlations and multiple null results.
3. Genes involved in translation/transcription are known to be enriched near oriC and are also likely to be highly expressed and growth responsive. It would strengthen the manuscript to show whether the main patterns persist after excluding or controlling for major functional classes.
4. Mapping candidate promoters to genes and retaining only the strongest promoter per gene may not adequately capture native promoter architecture. Also, assigning zero activity in the missing medium for promoters detected in only one medium could bias the analysis. These limitations should be discussed

Minor:

1. Mapping genes across strains by common name may be error-prone. Provide a bit more detail on how ambiguities were handled.
2. The final paragraph of the intro is very vague. I would appreciate a more substantial summary of the dataset and results.
