# Supplementary material for: Bacterial chromosomal gene positioning is likely shaped by selection on both mean and growth-dependent expression
Source: PLoS Genet. 2026 Sep 21;22(9):e1012316. doi: 10.1371/journal.pgen.1012316 (PMC13607872; doi:10.1371/journal.pgen.1012316)
Supplement: Supplementary file 12 [file pgen.1012316.s014.pdf]

Response to the editor and reviewers

We thank the editor and two reviewers for their valuable comments, which have helped improve the clarity and rigor of our manuscript. We performed all additional analyses suggested by the editor and reviewers and discussed limitations of our study. Below please find our point-to-point response in blue.

Editor

The manuscript is interesting but I believe that the authors should conduct additional analyses. In their current analyses, the authors do not distinguish core genes from accessory genes. Because accessory genes are often gained and lost through horizontal transfer, their expression levels are likely less impacted by their position on the chromosome. In contrast, core genes are long-term "residents" of the chromosome and their expression levels are more likely to be shaped by their position. I think that the author should further analyze their expression data for core genes and accessory genes independently as it might reveal clearer trends.

We followed the editor's insightful comment to build a model including a pangenome class × gene position interaction term (Model 2 in Materials and Methods). Analysis of this model suggests that core genes have a significantly stronger signal for the replication-dose effect and a significantly weaker signal for the mean promoter strength gradient along the chromosome than accessory genes in the *E. coli* consensus data (S7 Fig). However, the differences between core genes and accessory genes shrink and become non-significant when translation & transcription (TT) genes are excluded from the analysis, suggesting that the observed differences by pangenome class are mainly driven by TT genes. These results have been added to the manuscript (Lines 313-332 and 350-355).

Reviewer 1

In this manuscript, Yuan and Zhang use published datasets of bacterial growth rate and protein abundance to try to establish how gene positioning along the bacterial chromosome might be influenced by the optimal amount of each protein during cell growth. They use eight datasets from 3 different species.

The authors present two hypotheses. The mean expression hypothesis (MEH) states that the mean of the ideal amount of each protein across different environments explains the position of the genes in the bacteria chromosome, with the ones closest to the origin having higher expression. The growth-dependent expression hypothesis (GEH) states that genes closer to the origin have higher expression in growth conditions that lead to higher growth rates, while the opposite is true for genes closer to the terminus.

In the end, the authors find that both hypotheses are supported by the experimental data, at least in *E. coli*. However, even the combination of both fails to fully explain gene positioning across the bacterial chromosome. Moreover, the authors show that promoter strength also varies with gene position in E. coli, which might bias the interpretation of their results.

In my opinion, the main limitation of the study is the assumption that the gene expressions that are experimentally determined are the optimal ones. This assumption might not be ideal, because

the bacteria they are analyzing have spent the vast majority of their evolutionary time in nature, as opposed to in a lab, where the data comes from. The expression of some (most?) genes might have evolved to be optimal in an environment or environments that are very different from the ones analyzed. The authors acknowledge this, and do an okay job of justifying this assumption. Despite this limitation, and others explained by the authors in the discussion, I think the study is interesting enough for publication.

We thank the reviewer for summarizing our findings and for the overall positive evaluation. We agree with the reviewer and have already discussed in the manuscript the limitation of our study in assuming that expression has been optimized for all genes (Lines 378-394).

Here are some minor changes the authors can make that will improve the manuscript.

1) Figure 1 does a great job of summarizing both starting hypotheses. However, I think it would be best to use the same example genes for both (either gene A, B and C or X, Y, and Z), as the two different analyses are done to the same set of genes.

We thank the reviewer for this suggestion. Figure 1 now uses the same genes for both hypotheses.

2) The almost complete lack of significance results in the *B. subtilis* datasets is a little bit puzzling. Do the authors have any plausible explanation for this? If so, please add it to the discussion

One plausible explanation is that the *B. subtilis* consensus data have a limited number of conditions (15 conditions vs. 62 conditions in *E. coli*), making the k estimate less reliable, which could result in a weaker signal of the replication-dose effects. Additionally, the *B. subtilis* consensus data have the fewest genes (n = 951 for *B. subtilis* consensus data; n = 1700 for *E. coli* consensus data; and n = 1443 for *Zhu V. natrigens* data) and thus the statistical power is relatively low. However, we cannot exclude the possibility that the replication-dose effects are truly weaker in *B. subtilis* than in the other two species. We have added the above discussion to the manuscript (Lines 426-433).

3) I like the plots in figure 4, even if they are noisy. I think that it will be useful to make a similar graph including all environmental conditions and put it in figure 2 (or in a new supplemental), either for all datasets or just for the consensus datasets. I find this type of graph more intuitive than just plotting the correlation coefficient. Similarly, a graph with k in the y-axis and gene positioning in the x-axis would also be useful.

We have followed the reviewer's suggestion to add two supplementary figures (S1 Fig and S5 Fig) showing the gradients of $\overline{\ln E}$ and k along the chromosome for each individual dataset.

4) Fig S4 is mentioned before S2 and S3. This is likely due to methods being at the end of the manuscript and not before the results section.

The supplementary figures are now mentioned in the correct order.

5) The analysis related to figure S5 should be moved to the results section, since it is a result

After careful consideration of the suggestion, we decided to keep this supplementary figure in Discussion, because it is not related to our main results but is related to potential future studies. We are afraid that including it in Results would interrupt the presentation of our main findings.

6) The authors keep referring to the replication-dose effect, but never define it or cite any relevant literature. For instance, the authors might want to cite and briefly discuss PMID38267581.

We have now added a definition of the replication-dose effect. We have cited the reference PMID38267581 in Discussion (Line 459) because it is more relevant to the explanation of mechanisms of gene expression deviations from the replication-dose effect.

7) The authors should discuss that the likelihood of a gene switching positions in the chromosome is much less likely that a point mutation that leads to a change in promoter strength, which might explain the small predictive power of the two hypotheses.

We have added this point (Line 446).

8) The authors should tone down some of their conclusions. For instance, from the abstract: "We observe a significant decrease in both mean expression and expression slope from *oriC* to *ter*, consistent with both MEH and GEH". This is only true for *E. coli*. There are a few other sentences like this when it should be explained that both hypotheses are supported only in *E. coli*.

We agree. We have toned down our conclusions and have clarified specific datasets/species or fraction of datasets when a conclusion is supported by only a subset of the data (e.g., see Abstract).

Reviewer 2

This manuscript examines whether bacterial chromosomal gene positioning is shaped by selection on mean expression (MEH) and/or growth-dependent expression (GEH). To do this, the study examines eight multi-condition proteomic/ribosome-profiling datasets from three bacterial species, along with an *E. coli* promoter dataset, the authors report that both mean expression and expression slope with growth rate tend to decrease from oriC to ter. They conclude that both MEH and GEH contribute to gene positioning, although with modest explanatory power.
The manuscript addresses an interesting and important question in bacterial genome organization. The conceptual framing is clear, the compilation of multiple public datasets is a strength, and the conclusion that any effect is modest is appropriately cautious. The promoter strength analysis is also potentially valuable.

We thank the reviewer for the overall positive evaluation.

That said, I have several substantive concerns that should be addressed before publication.

Major:
1. The manuscript should more clearly distinguish that the findings are consistent with adaptive positioning rather than providing direct evidence that selection shaped positioning.

We agree and have toned down the statements regarding selection on gene positioning, including changing "is shaped by selection" to "is likely shaped by selection" in the title.

2. With such a small sample size of transcriptomic data where only ~half had significant results, it isn't appropriate to assume results are globally applicable. A call to apply this analysis to more datasets from more varied bacterial species to better understand trends should be included in the discussion. For example, statements such as "clear evidence for MEH/GEH" feel too strong considering the modest correlations and multiple null results.

We agree that whether our conclusions can be generalized to other species and datasets is unknown. We have added this caveat to Discussion and have toned down our conclusions (Lines 433-439).

3. Genes involved in translation/transcription are known to be enriched near oriC and are also likely to be highly expressed and growth responsive. It would strengthen the manuscript to show whether the main patterns persist after excluding or controlling for major functional classes.

We thank the reviewer's insightful suggestion. We have followed the comment to test the remaining positional gradients in $\overline{\ln E}$ and k after excluding translation and transcription genes. We found the effects to be substantially weakened after excluding TT genes (S9 Fig). To formally test the effect of TT genes, we added a test and found that TT genes make disproportionately large contributions to the positional gradients in both $\overline{\ln E}$ and k in all datasets analyzed (newly added Fig 5). The finding that positional gradients persist in several datasets after excluding TT genes suggests that non-TT genes also contribute to the positional gradients, but their per-gene contribution is significantly less than TT genes. This is consistent with the hypothesized stronger selection on gene position for TT genes than other genes. We have added the new analysis and results to the manuscript (Lines 153-159, 334-349, and 601-616).

4. Mapping candidate promoters to genes and retaining only the strongest promoter per gene may not adequately capture native promoter architecture. Also, assigning zero activity in the missing medium for promoters detected in only one medium could bias the analysis. These limitations should be discussed

We agree that using the strongest promoter strength and assigning 0 activity to the promoters undetected in a medium are limitations in the promoter strength estimation procedure. We have added these limitations to Discussion (Lines 471-481).

Minor:
1. Mapping genes across strains by common name may be error-prone. Provide a bit more detail on how ambiguities were handled.

We agree that mapping genes by common names is potentially error-prone and ambiguous, especially when one-to-multiple or multiple-to-multiple mapping is involved. We have now mapped genes to unique locus tags of the reference strains (strain MG1655 for *E. coli* and strain 168 for *B. subtilis*) and used the annotation data of the mapped locus tags for all downstream analyses. We had to map genes to the reference locus tag by gene common name for the Li dataset because gene common name is the only gene identifier available for ribosomal profiling data in the dataset. For all other datasets, we used gene common name only as a fallback when mapping by other identifiers fails (0.075% of gene mapping uses the common name fallback). We also required one-to-one mapping and excluded all ambiguous mapping. These methodological improvements slightly increased the number of genes that could be analyzed. These details have been added to the manuscript (Lines 494-507).

2. The final paragraph of the intro is very vague. I would appreciate a more substantial summary of the dataset and results.

We have specified the main datasets used and added r^2 in the beta regression models to make the paragraph more concrete (Lines 160-165).
