# Supplementary material for: Bacterial chromosomal gene positioning is likely shaped by selection on both mean and growth-dependent expression
Source: PLoS Genet. 2026 Sep 21;22(9):e1012316. doi: 10.1371/journal.pgen.1012316 (PMC13607872; doi:10.1371/journal.pgen.1012316)
Supplement: Supplementary file 13 [file pgen.1012316.s011.docx]

In this manuscript, Yuan and Zhang use published datasets of bacterial growth rate and protein abundance to try to establish how gene positioning along the bacterial chromosome might be influenced by the optimal amount of each protein during cell growth. They use eight datasets from 3 different species.

The authors present two hypotheses. The mean expression hypothesis (MEH) states that the mean of the ideal amount of each protein across different environments explains the position of the genes in the bacteria chromosome, with the ones closest to the origin having higher expression. The growth-dependent expression hypothesis (GEH) states that genes closer to the origin have higher expression in growth conditions that lead to higher growth rates, while the opposite is true for genes closer to the terminus.

In the end, the authors find that both hypotheses are supported by the experimental data, at least in *E. coli*. However, even the combination of both fails to fully explain gene positioning across the bacterial chromosome. Moreover, the authors show that promoter strength also varies with gene position in E. coli, which might bias the interpretation of their results.

In my opinion, the main limitation of the study is the assumption that the gene expressions that are experimentally determined are the optimal ones. This assumption might not be ideal, because the bacteria they are analyzing have spent the vast majority of their evolutionary time in nature, as opposed to in a lab, where the data comes from. The expression of some (most?) genes might have evolved to be optimal in an environment or environments that are very different from the ones analyzed. The authors acknowledge this, and do an okay job of justifying this assumption.

Despite this limitation, and others explained by the authors in the discussion, I think the study is interesting enough for publication.

Here are some minor changes the authors can make that will improve the manuscript.

1) Figure 1 does a great job of summarizing both starting hypotheses. However, I think it would be best to use the same example genes for both (either gene A, B and C or X, Y, and Z), as the two different analyses are done to the same set of genes.
2) The almost complete lack of significance results in the *B. subtilis* datasets is a little bit puzzling. Do the authors have any plausible explanation for this? If so, please add it to the discussion
3) I like the plots in figure 4, even if they are noisy. I think that it will be useful to make a similar graph including all environmental conditions and put it in figure 2 (or in a new supplemental), either for all datasets or just for the consensus datasets. I find this type of graph more intuitive than just plotting the correlation coefficient. Similarly, a graph with k in the y-axis and gene positioning in the x-axis would also be useful.
4) Fig S4 is mentioned before S2 and S3. This is likely due to methods being at the end of the manuscript and not before the results section.
5) The analysis related to figure S5 should be moved to the results section, since it is a result
6) The authors keep referring to the replication-dose effect, but never define it or cite any relevant literature. For instance, the authors might want to cite and briefly discuss PMID38267581.
7) The authors should discuss that the likelihood of a gene switching positions in the chromosome is much less likely that a point mutation that leads to a change in promoter strength, which might explain the small predictive power of the two hypotheses.
8) The authors should tone down some of their conclusions. For instance, from the abstract: “We observe a significant decrease in both mean expression and expression slope from *oriC* to *ter*, consistent with both MEH and GEH”. This is only true for *E. coli*. There are a few other sentences like this when it should be explained that both hypotheses are supported only in *E. coli*.
